# Chiral Polaritonics: Analytical Solutions, Intuition, and Use

**DOI:** 10.1021/acs.jpclett.3c00286

**Published:** 2023-04-13

**Authors:** Christian Schäfer, Denis G. Baranov

**Affiliations:** † MC2 Department, Chalmers University of Technology, 41258 Gothenburg, Sweden; ‡ Center for Photonics and 2D Materials, Moscow Institute of Physics and Technology, Dolgoprudny 141700, Russia

## Abstract

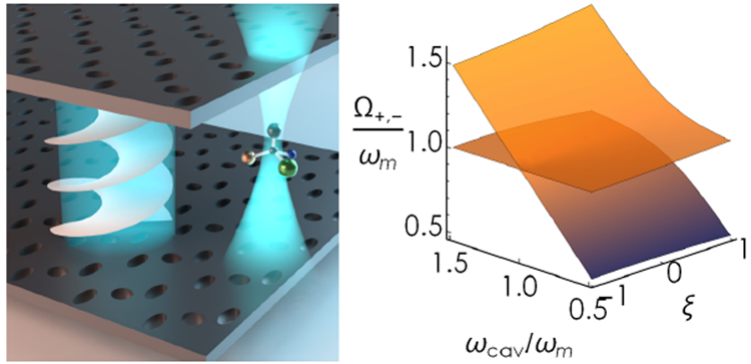

Preferential selection of a given enantiomer over its chiral counterpart has become increasingly relevant in the advent of the next era of medical drug design. In parallel, cavity quantum electrodynamics has grown into a solid framework to control energy transfer and chemical reactivity, the latter requiring strong coupling. In this work, we derive an analytical solution to a system of many chiral emitters interacting with a chiral cavity similar to the widely used Tavis–Cummings and Hopfield models of quantum optics. We are able to estimate the discriminating strength of chiral polaritonics, discuss possible future development directions and exciting applications such as elucidating homochirality, and deliver much needed intuition to foster the newly flourishing field of chiral polaritonics.

Coupling between two harmonic oscillators, either of classical or quantum origin, leads to a hybridization and the creation of new quasi-particle states if the coupling strength exceeds all of the decay and decoherence rates in the combined system. A common representative of such a system is the interaction between an energetically isolated electromagnetic mode and a set of quantum emitters, such as molecules. The associated quasi-particle states are referred to as polaritons and possess mixed light and matter characteristics, which opens a toolbox with enormous versatility.^1−15^ Polaritons of various flavors have been used or proposed as a path to enhanced charge and excitation transfer,^16−22^ modify chemical reactivity,^23−38^ and alter a system’s state and response to external stimuli,^39−48^ to name only a few.

So far, most experimental and theoretical efforts in this field have focused on coupling optical cavities with either linearly or circularly polarized electronic transitions of various quantum emitters. This is perfectly justified by the fact that in the visible and infrared ranges the interaction of light with electronic and vibrational transitions is dominated by the electric dipole term of the Hamiltonian. Nevertheless, there are examples of media that exhibit resonances with a non-negligible magnetic transition dipole moment. One such practically relevant example is presented by the class of chiral media.^49−51^

A geometrical shape in 3D space is called chiral if it cannot be aligned with its mirror image by a series of rotations and translations.^52^ The chirality occurs on various scales ranging from the shapes of galaxies down to drug and biomolecules. In particular, the latter receives a steady stream of attention in the ongoing quest for new, safer, and affordable ways to design chemical complexes and drugs.^53,54^ It is then intuitively pivotal for its success to develop a solid understanding of relevant processes and a wide range of readily usable techniques that allow separation or discrimination of the two enantiomers of a chiral structure. While recent years have shown major progress in this field,^55,56^ including, among others, optical force-assisted separation,^57,58^ the most widely used chemical strategies such as (re)crystallization^59^ can be cumbersome and often require highly specified approaches for each individual compound.

The interaction of chiral matter with circularly polarized electromagnetic fields leads to the effect of circular dichroism, which underlies numerous methods for distinguishing molecular enantiomers.^60^ However, those interactions are usually weak and can be well understood without the need to consider a correlated motion between light and matter. If and how strongly the light–matter interaction can aid those challenging tasks remained largely unclear thus far.

While chiral polaritonics is still in its infancy, recent theoretical work is beginning to explore this question. Mauro et al. investigated the optical features of a single-handedness cavity loaded with a Pasteur medium by using classical electromagnetism.^61^ Riso et al. studied changes in the correlated ground state of single (or few) realistic molecules minimally coupled to an amplified chiral mode.^62^ Related to, yet distinct from, chirality are approaches that involve optical spin–orbit coupling^63,64^ or cavity designs that break time-reversal symmetry.^13,65^

In this letter, we provide an analytical solution and much needed intuition that will be of good use for the future development of chiral polaritonics. Starting from nonrelativistic quantum electrodynamics (QED), we derive the quantized electromagnetic fields supported by a single-handedness chiral cavity^66^ and couple them to a large set of chiral emitters as illustrated in Figure 1. The resulting Hamiltonian can serve as a starting point for any kind of *ab initio* QED.^13,67−71^ Here, we focus on a simplified model that allows for an analytical solution that illustrates the physical playground, potential, and forthcoming challenges of chiral polaritonics. We focus our discussion on molecular systems but emphasize that the derived models and conclusions can be transferred to other resonant chiral systems, such as meta-atoms. In contrast to previous approaches,^55,56,72−74^ the strong coupling to a chiral cavity allows one to reach sizable interaction strength even in the absence of any pumping field. The model derived here paves the way to invigorating an entirely new research domain.

**Figure 1:**
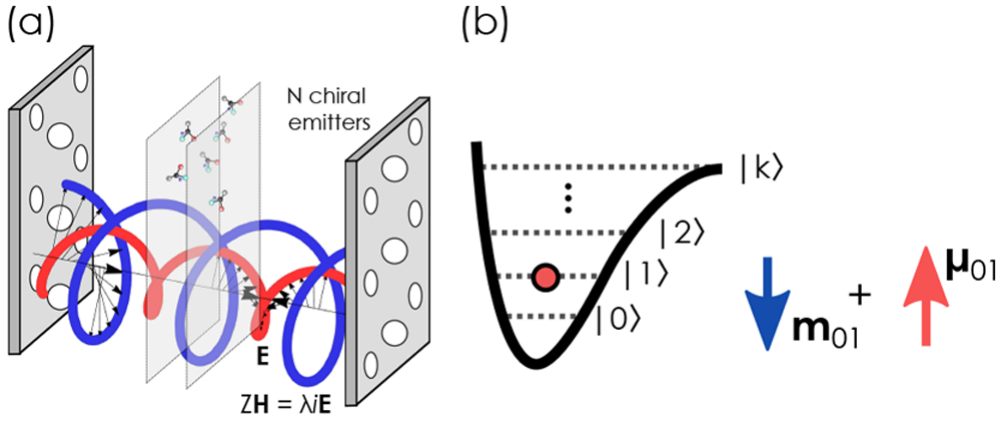
(a) Illustration of the system under study. *N* identical chiral (quantum) emitters interact with the electromagnetic field of a chiral standing wave. A chiral standing wave is formed between two handedness-preserving metasurface mirrors. (b) Model of a single chiral emitter as a generic many-level system. Chiral emitters, which represent molecules, biological structures, or plasmonic meta-atoms, are modeled as simplified multilevel systems whose transitions are quantified by collinear electric and magnetic dipole moments.

We start our derivation from the nonrelativistic limit of QED in Coulomb gauge. Using the Power–Zienau–Wooley transformation and expanding the multipolar light–matter interaction to second order introduces magnetic dipolar couplings and electric quadrupole terms^75−78^ according to
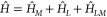
where we differentiate between the *N*_*M*_ electronic (*q*_*i*_ = −*e*) and nuclear (*q*_*i*_ = *eZ*_*i*_) charges plus longitudinal Coulomb interaction among them constituting the molecule, and intermolecular Coulomb interactions
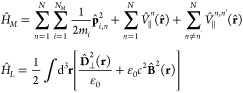
The interaction *H*_*LM*_ up to magnetic order takes the form
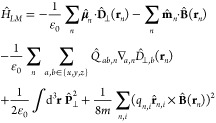
1with the canonical particle momentum , the total transverse polarization , the electric dipole moment  and quadrupole *Q*_*ab*_, and the magnetic dipole , which we extended by the spin contribution mediated via the *g* factor *g*_*i*_.^79,80^ All positions are defined relative to each individual molecular center of mass. Importantly, the multipolar form introduces the displacement field  as the canonical momentum to the vector potential. The last term in eq 1 may be referred to as diamagnetic interaction and can be written in the tensorial form  with .

Let us briefly comment on the relevance of the self-interaction contributions. The magnetic  and electric  self-polarization terms ensure gauge invariance and guarantee the stability of the correlated system.^76,81^ A common simplification is to assume that the molecules are well separated. In this dilute limit and when all photonic modes are considered, the intermolecular Coulomb interactions cancel perturbatively with the intermolecular contributions arising from  such that only retarded intermolecular interactions via the photonic fields remain.^76,82^ However, when the number of photonic modes is truncated, as is commonly done for polaritonic systems, this intuitive result no longer holds. If the intermolecular contributions are neglected nevertheless, then one arrives at the widely used Dicke model that falsely predicts a transition into a superradiant phase, while the associated model in the Coulomb gauge does not exhibit such a transition.^83^ Under which conditions a phase transition for more realistic systems could appear and what characterizes such a transition are still matters of active debate.^42,84−88^ We provide a derivation similar to the common Dicke model in the SI and focus on the following in the development of a nonperturbative chiral model.

The electromagnetic fields follow the generic mode expansion
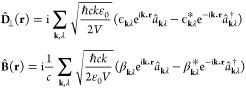
where *V* is the cavity mode volume, ϵ_**k**,λ_ and β_**k**,λ_ are the electric and magnetic field unit polarization vectors, **k** labels wave vectors, and λ labels polarization states.

Although Maxwell’s equations in free space admit solutions in the form of chiral photons, in this case, both handednesses coexist at the same time. Contrary to one’s intuition, illuminating an ordinary Fabry-Pérot cavity with circularly polarized light does not address this problem.^13,89^ However, by using cleverly designed asymmetric “single-handedness” cavities,^66^ it is possible to engineer pure chiral electromagnetic fields with only one handedness. Although such single-handedness cavities have been studied to date only theoretically, there is notable progress in the experimental realization of asymmetric handedness-preserving mirrors.^90^

In a right-handed (left-handed) monochromatic wave propagating through free space, the magnetic field is π/2 behind (ahead) the electric field everywhere in space *Z***H**(**r**; ω) = −i*λ***E**(**r**; ω), where  is the free space impedance and λ is the eigenvalue of the helicity operator,^91^ which takes values of +1 and −1 for LH and RH fields, respectively. Only a subset of modes will adhere to the conditions that are imposed by the boundary conditions of the chiral cavity; it should be noted that the electromagnetic fields are not zero at the mirror surfaces. Modes with opposite handedness, i.e., those not supported by the cavity boundary conditions, propagate freely through the idealized system and retain their free-space spectrum. We will focus our discussion on the confined modes responsible for (ultra)strong coupling, while the free-space modes with mismatched handedness could be accounted for by typical, but now chiral, system-bath descriptions.

A planar optical cavity, such as the one described in ref (66), supports a continuous spectrum of resonant states that can be labeled by their in-plane momenta **k**_∥_. Cavity fields maintain their single-handedness quality for a substantial range of in-plane wave vectors (incident angles).^66^ For simplicity, we will illustrate only coupling of a single standing wave (**k**_∥_ = 0) to the chiral emitters and refer the interested reader to the SI for a generalized discussion. The chiral standing wave is the superposition of two counter-propagating circularly polarized plane waves of the same handedness. Assuming the axis of the cavity to be pointed along the *z* direction and considering a vertical standing wave with **k** = ±*k***e**_*z*_, the displacement field  of a LH/RH standing wave is  with  and .

With these simplifications, the displacement field of a chiral standing wave takes the form
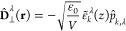
where  is the *z*-dependent polarization vector of the chiral standing wave and the canonical coordinates are  and . Notice that the left- and right-handed polarization vectors are orthogonal only in the spatially averaged sense. Recalling the relation between the electric and magnetic fields of a chiral field, β_**k**,λ_ = −i*λϵ*_**k**,λ_, we obtain the magnetic field of a chiral standing wave as .

The standing chiral fields satisfy Maxwell’s equation and contribute with *ck* = ω_*k*_ a photonic energy of  for a given handedness. The extraordinary consequence is now that the standing field in an empty cavity will feature chiral quantum fluctuations; i.e., for each Fock-state |*n*⟩, the optical chirality density  reduces to *C*_*n*_(**r**, ω) = *λℏω*_*k*_*k*/4*V*. Also, a dark chiral cavity will influence the ground and excited states of matter located within it. In the following, we introduce a series of simplifications and derive an analytical solution to the combined system of many chiral molecules coupled to the chiral cavity.

Let us briefly describe the derivation of the analytical solution and its underlying models; a detailed version can be found in the SI. For negligible intermolecular Coulombic interactions, i.e., , the molecular component to the Hamiltonian becomes diagonal  in the many-body eigenstates |*k*⟩. Expanding all transition elements in this eigenbasis would, in principle, allow for numerical solutions of the (ultra)strongly coupled system. The interested reader might refer to the area of *ab initio* QED.^92^ Here, we focus on simplified models that provide analytical solutions.

The self-magnetization term mediated via  will be assumed to be purely parametric  as it otherwise obstructs the Hopfield diagonalization scheme. The self-magnetization ensures gauge invariance and should be expected to play an important role in more sophisticated *ab initio* approaches. We define the dressed photonic frequency  which is related via the sum rule  to the eigenvalues *E*_*m*_ and eigenstates |*m*⟩.

In order to provide analytical solutions, we will limit ourselves in the following to either two-level systems  or harmonic oscillators . Both approaches are widely used, but we will focus ultimately on the harmonic representation as it allows us to access ultrastrong correlation in the analytical solution. Our molecules are neutral such that we disregard permanent dipole moments for brevity.

The relationship between the transition dipole moments has the generic form
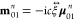
2where ξ⃡ is the product of a 3D rotation and scaling (SI). For brevity, we are going to limit our analysis to molecules with collinear transition dipole moments and refer the reader to the SI for generalization. In this scalar case, ξ = +1 and ξ = −1 describe ideal LH and RH emitters, respectively.^51^ This allows us to combine the electric dipole, electric quadrupole, and magnetic dipole into a single compact expression when **μ**_10_^*n*^ = **μ**_01_^*n*^ and *Q*_*ab*,*n*_^10^ = *Q*_*ab*,*n*_^01^, **Q**_*n*_ = *Q*_*ab*,*n*_^10^∇_*a*_**e**_*b*_. From here on, we are left to follow two different but equally popular directions that we detail in the following.

A convenient and widely used approximation in quantum optics is to assume that the molecular basis consists of merely two states motivated by the anharmonicity of excitonic transitions. For very large coupling strength, there is little reason to believe that the complicated multilevel structure of chiral molecules is well captured by only a single excitation. Let us assume for a moment that we would limit ourselves to this parameter regime. The excitation spectrum is then approximated by a single excitation of energy *ℏω*_*m*_ which is commonly transferred into a Pauli spin basis |1⟩⟨0| → σ^+^. If we further discard the self-polarization term and counter-rotating terms , then we obtain the strongly simplified chiral Tavis–Cummings Hamiltonian
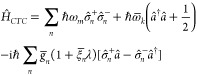
The chiral coupling is encoded via the effective interaction strength proportional to . A chiral emitter that features the same (opposite) handedness as the cavity will couple more strongly (weakly) to the mode. In the extreme case that , the mismatched enantiomer will entirely decouple from the mode. The above chiral Tavis–Cummings model can be solved analogously to the standard Tavis–Cummings model by introducing collective spin operators.

Once we approach the ultrastrong coupling domain, many excitations of the chiral molecule will contribute to the renormalization of transitions. A possible alternative is the harmonic approximation, by analogy to Hopfield,^93^ in which we identify the excitation structure with that of a harmonic oscillator. The resulting Hamiltonian takes the form of *N* + 1 coupled harmonic oscillators and can be solved via Hopfield diagonalization (detailed in the SI). The Hopfield solution is known to provide accurate predictions for effectively bosonic systems, such as vibrations^94^ and intersubband transitions.^95^ We find that the qualitative predictions of our Hopfield model are consistent with available *ab initio* calculations. (See the SI and the following text.) It should be noted that chiral Tavis–Cummings and Hopfield models provide consistent predictions for the first polariton manifold under strong coupling as  in the single-excitation space.

Assuming identical (but distinguishable) molecules and a homogeneous in-plane distribution, it is convenient to introduce collective molecular operators , where **k**_∥_ is the in-plane momentum of the matter excitation. We discard the decoupled dark states and assume zero in-plane momentum for brevity; an extended discussion can be found in the SI. Under those approximations
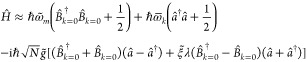
3where ,  and  are the renormalized effective excitation energy, coupling strength, and chirality factor.

Equation 3 is diagonalized by following the standard Hopfield^93,95^ procedure, i.e., defining the polaritonic operator  that fulfills the eigenvalue equation  with the normalization condition |*x*|^2^ – |*y*|^2^ + |*z*|^2^ – |*u*|^2^ = 1. We obtain the polaritonic frequencies as real and positive solutions
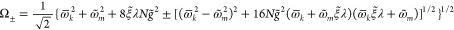
4with eigenvalues *E*_±_ = *ℏ*Ω_±_ + *E*_*vac*_, where *E*_*vac*_ = *ℏ*(Ω_+_ + Ω_–_)/2, up to an arbitrary constant that is independent of the handedness of the emitter.

As illustrated in Figure 2(a), an ensemble of ideal chiral emitters featuring the opposite handedness  compared to the LH cavity mode effectively decouples from the cavity. Switching the cavity handedness would therefore allow one to open and close the avoided crossing- and control-associated conical intersections. This is an intuitively expected result: an ensemble of LH molecules couples to the LH photonic mode, whereas an ensemble of equivalent RH molecules becomes transparent for the same optical mode. The cross-section of the full plot at ξ = 0 yields the familiar picture of “traditional” polaritons with electric-dipole-mediated coupling.^95^ Furthermore, the photonic and matter polariton fractions exhibit a gradual transition from the regime of hybridized eigenstates at ξ̃λ = 1 to the uncoupled regime at ξ̃λ = −1, when the two eigenstates represent bare optical and matter excitations. Our simple Hopfield solution recovers the promising feature that the vacuum coupling in chiral cavities can be used to discriminate between two enantiomers.

**Figure 2:**
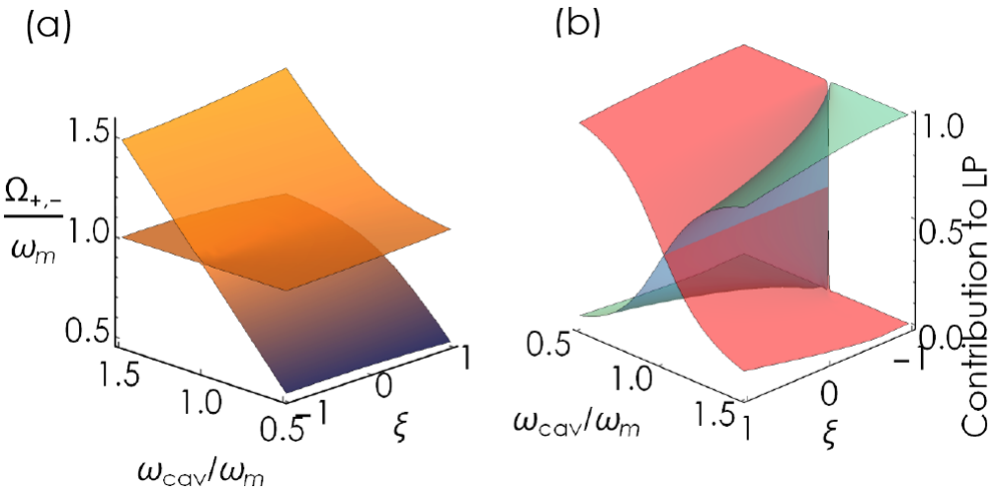
(a) Polaritonic eigenvalues Ω_±_ of the chiral Hopfield model with an LH cavity mode (λ = 1) for *N* = 100 as a function of the cavity frequency ω_*k*_ and chiral factor ξ. The eigenvalues are calculated with typical values for optical transitions in dye molecules,^60^**Q** = χ_*i*,*j*_^*m*^ = *z* = 0, and the fundamental coupling strength of  (a.u.). (b) Hopfield coefficients (light blue, photonic; red, matter) for the lower polariton for the same system as in panel (a).

Importantly, the cavity distinguishes only the chiral component, i.e., the parallel projection of **μ**. This can be easily seen by extending our discussion to consider general bi-isotropic media and performing an angular average (SI). Let us recall that, in addition to the illustrated bright states, *N* – 1 dark states exist that remain largely unchanged. Irrespective of this large imbalance in number, polaritonic chemistry has demonstrated that measurable changes in energy transfer and reactivity appear (see introduction). The critical condition is strong coupling, i.e., precisely what the chiral cavity is able to selectively control for each enantiomer.

A vast majority of widely used molecular systems exhibit extremely weak chirality factors ξ ≪ 1, rendering it challenging to separate left- and right-handed enantiomers to high fidelity. Chiral polaritonics can serve this purpose as the collective interaction results in a  scaling of the coupling strength, thus increasing the selectivity. Figure 3 illustrates the difference in upper and lower polaritonic eigenfrequencies (blue) between left- and right-handed enantiomers for typical dye molecules^60^ with a small and conservative estimate of ξ ≈ 3.712 × 10^–5^ for the chirality factor.

**Figure 3:**
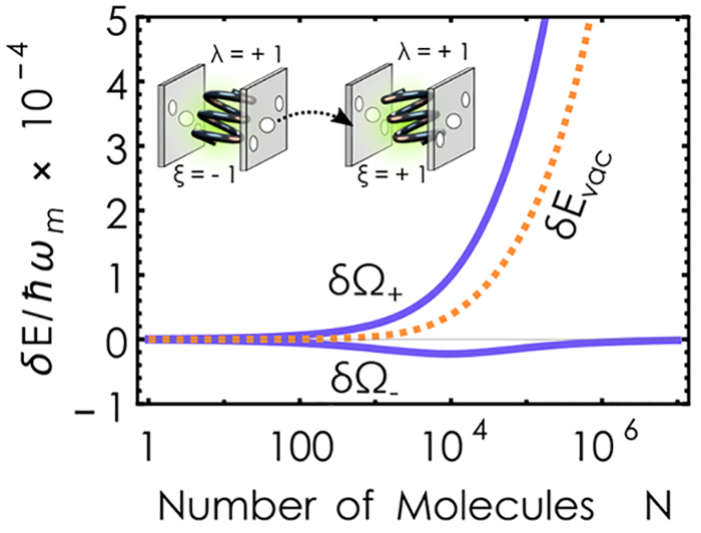
Normalized difference between the polaritonic excitation energies δΩ_±_ = Ω_±_^ξ=+1^ – Ω_±_^ξ=–1^ (blue solid line) and the correlated ground state *δE*_*vac*_ = *E*_*vac*_^ξ=+1^ – *E*_*vac*_^ξ=–1^ (orange dotted line) of left- and right-handed chiral dye molecules inside an LH chiral cavity. We have chosen common values for dye molecules,^60^ the resonant condition ω_*k*_ = ω_*m*_, and  in atomic units for the chiral Hopfield model. For comparison, a molecular concentration of 1 mol/L represents approximately 10^3^ molecules in the chosen volume. The actual number of collectively coupled emitters under experimental conditions is usually unknown and is estimated based on simplified models, such as the one presented here.

For large *N*, the interacting system enters into the ultrastrong coupling domain in which the combined light–matter ground state is no longer separable. The relative energy difference between LH/LH and RH/LH ground states is shown in Figure 3 (orange dotted line) as a function of the number of molecules, *N*. Clearly, the correlated ground and excited states illustrate a quick increase in the discriminating effect—the center point of chiral polaritonics. The ground-state discrimination scales linearly in *N* for moderate coupling and continues to scale as  in the deep ultrastrong coupling domain (SI). Both limits are consistent with the observation by Riso et al.^62^

The small value of ξ for typical molecules translates into an overall small eigenvalue difference that scales on resonance approximately with . While  can reach a sizable fraction of the excitation energy, a major limitation to be fought here is the commonly weak chirality. Polaritonic chemistry will require a noticeable difference in the effective coupling with respect to the enantiomers. In order to leverage chiral polaritonics for enantiomer selectivity, it seems essential to either magnify the magnetic components or establish a protocol that can exploit the small energetic differences.

The latter follows closely the still open question of the origin of homochirality; i.e., how could a minute energetic imbalance between the enantiomers result in the real-world dominance of a given handedness.^96^ Among the frequently discussed options are autocatalytic processes that turn a small imbalance into a substantial excess.^97^ Chiral polaritonics not only could explore a similar path but also could serve as a sensitive framework to further elucidate the origin of homochirality.

The former approach, on the other hand, would propose the design of a cavity that would compensate for the small ξ̃. The discriminating factor 1 + ξ̃λ in our purely transversal cavity is bound by the small size of ξ̃ as |λ| = 1 is fixed by *Z***H** = −iλ**E**. The latter, however, no longer holds in subwavelength cavities where the field has a significant longitudinal component. In (nonchiral) plasmonic nanocavities, for example, |*Z***H**| ≪ |**E**|. Thus, the challenge could be addressed by designing a compact chiral nanocavity, whose quasi-normal mode is dominated by the longitudinal magnetic field, |*Z***H**| ≫ |**E**|, and maintains chiral character expressed by a nonzero local chirality density *C*(**r**).

Finally, we note that the developed Hamiltonian models are not restricted to molecular systems. Transferring our conclusions merely requires that electric and magnetic dipolar contributions dominate the light–matter interaction and that the spectral structure of the cavity and of the emitter(s) is consistent with the limitations of the chiral Hopfield or Tavis–Cummings model. Especially relevant alternatives include resonant plasmonic meta-atoms and metasurfaces^89,98,99^ made of achiral media but retaining geometrical chirality.

To conclude, we developed a new analytical model describing the nonperturbative interaction of an ensemble of chiral molecules with a common chiral optical mode, i.e., a resonator that supports only optical modes with a given handedness. The model illustrates that a chiral cavity can be used to selectively couple to molecules of a specific handedness and thus provides a means to discriminate enantiomers from a racemic via the versatile tool box of polaritonic chemistry and cavity QED. Such a chiral discriminating effect can be observed in all eigenstates of the strongly hybridized light–matter system and exists in the absence of any pumping, i.e., in the dark cavity. How strong left- and right-handed enantiomers can be distinguished is proportional to . While  can become sizable, the degree of chirality typically satisfies ξ ≪ 1, which currently limits the capability for chiral recognition. Possible strategies to exploit field enhancement techniques^60^ in combined optical and plasmonic systems^100^ might pave the way to enhanced recognition capabilities. It should be noted that this simple, accurately controllable, and easily realizable system breaks a discrete symmetry with possibly wide and yet unforeseen ramifications. Chiral polaritonics contributes, even at this early stage, an exciting perspective to further elucidate homochirality. The intuitive analytical model and perspective put forward in this letter will foster this new domain on the intersection of cavity QED, chiral chemistry, biology, and nanophotonics.

## Supporting Information Available

Discussion and illustration of standing chiral waves; extended derivation of the chiral Hopfield and Tavis–Cummings models; discussion of transition dipole moments and the influence of their generic alignment for chiral recognition; illustration of relevance for self-magnetization contributions; extension to modes with nonzero in-plane momentum *k*_*x*_ ≠ 0; derivation of a chiral Hopfield model under the assumption of canceling instantaneous intermolecular contributions; and a brief study of the consistency with *ab intio* calculations in the ultrastrong coupling domain (PDF)
Transparent Peer Review report available (PDF)
