# Supplementary material for: Chiral Polaritonics: Analytical Solutions, Intuition, and Use
Source: J Phys Chem Lett. 2023 Apr 13;14(15):3777–84. doi: 10.1021/acs.jpclett.3c00286 (PMC10123817; doi:10.1021/acs.jpclett.3c00286)
Supplement: Supplementary file 1 — jz3c00286_si_001.pdf [file jz3c00286_si_001.pdf]

Supplemental Information to Chiral Polaritonics: Analytic Solutions, Intuition and its Use

Christian Schäfer*,† and Denis G. Baranov*,‡

†*MC2 Department, Chalmers University of Technology, Sweden*

‡*Center for Photonics and 2D Materials, Moscow Institute of Physics and Technology, Dolgoprudny 141700, Russia*

E-mail: christian.schaefer.physics@gmail.com; denis.baranov@phystech.edu

Abstract

The Supplemental Information includes: Discussion and illustration of standing chiral waves, extended derivation of the chiral Hopfield and Tavis-Cummings models, discussion of transition dipole moments and the influence of their generic alignment for chiral recognition, illustration of relevance for self-magnetization contributions, extension to modes with non-zero in-plane momentum $k_x \neq 0$, derivation of a chiral Hopfield model under the assumption of cancelling instantaneous intermolecular contributions, brief study of the consistency with *ab intio* calculations in the ultra-strong coupling domain.

S1 Fields of a standing chiral wave

In the following we obtain explicit expressions for the electric and magnetic fields of a chiral standing wave describing the optical mode of a single-handedness optical cavity. We begin with the case of a standing wave formed by two counter-propagating circularly polarized

plane waves. The field of a monochromatic circularly polarized plane wave propagating through air in the positive direction of the z axis takes the form ($e^{-i\omega t}$ time dependence for the harmonic field is assumed):

$$\mathbf{E}^{\lambda}_{+z}(\mathbf{r}) = \frac{\mathcal{E}}{\sqrt{2}} \begin{pmatrix} 1 \\ i\lambda \\ 0 \end{pmatrix} e^{ikz}, \quad Z\mathbf{H}^{\lambda}_{+z}(\mathbf{r}) = -i\lambda \mathbf{E}^{\lambda}_{+z}(\mathbf{r}), \tag{S1}$$

where $\lambda = \pm 1$ denotes the handedness of the wave, $k = \omega/c$, and $\mathcal{E}$ has units of electric field. Correspondingly, the fields of a wave travelling in the negative direction of the z axis takes the form:

$$\mathbf{E}^{\lambda}_{-z}(\mathbf{r}) = \frac{\mathcal{E}}{\sqrt{2}} \begin{pmatrix} 1 \\ -i\lambda \\ 0 \end{pmatrix} e^{-ikz}, \quad Z\mathbf{H}^{\lambda}_{-z}(\mathbf{r}) = -i\lambda \mathbf{E}^{\lambda}_{-z}(\mathbf{r}). \tag{S2}$$

The fields of a "vertical" standing wave take the form:

$$\mathbf{E}^{\lambda}_{\mathbf{k}_{\parallel}=0} = \frac{\mathbf{E}^{\lambda}_{+z} + \mathbf{E}^{\lambda}_{-z}}{\sqrt{2}} = \mathcal{E} \begin{pmatrix} \cos kz \\ -\lambda \sin kz \\ 0 \end{pmatrix}, \quad Z\mathbf{H}^{\lambda}_{\mathbf{k}_{\parallel}=0} = -i\lambda \mathbf{E}^{\lambda}_{\mathbf{k}_{\parallel}=0}(\mathbf{r}). \tag{S3}$$

Now consider a pair of circularly polarized plane waves with a given handedness λ both propagating with a fixed in-plane momentum $\mathbf{k}_{\parallel}$ (in-plane with respect to the vertical axis of the cavity) and opposite vertical component of the wave vector $\pm k_z \hat{\mathbf{z}}$. Without loss of generality let us assume $\mathbf{k}_{\parallel} = k_x \hat{\mathbf{x}}$ with $k_x = k \sin\theta$ and $k_z = \pm k \cos\theta$. Electric fields take the form:

$$\mathbf{E}^{\lambda}_{+z,\mathbf{k}_{\parallel}}(\mathbf{r}) = \frac{\mathcal{E}}{\sqrt{2}} \begin{pmatrix} \cos\theta \\ i\lambda \\ -\sin\theta \end{pmatrix} e^{ik_z z + ik_x x}, \quad \mathbf{E}^{\lambda}_{-z,\mathbf{k}_{\parallel}}(\mathbf{r}) = \frac{\mathcal{E}}{\sqrt{2}} \begin{pmatrix} \cos\theta \\ -i\lambda \\ +\sin\theta \end{pmatrix} e^{-ik_z z + ik_x x} \tag{S4}$$

The z-dependent electric field of the combination of the two waves with the common $\mathbf{k}_\parallel$ takes the form:

$$\mathbf{E}^{\lambda}_{\mathbf{k}_\parallel}(\mathbf{r}) = \mathcal{E}\begin{pmatrix} \cos\theta\cos k_z z \\ -\lambda\sin k_z z \\ -i\sin\theta\sin k_z z \end{pmatrix} e^{ik_x x}. \tag{S5}$$

Magnetic field of the chiral standing wave with handedness λ follows from the electric field:

$$Z\mathbf{H}^{\lambda}_{\mathbf{k}_\parallel} = -i\lambda\mathbf{E}^{\lambda}_{\mathbf{k}_\parallel}. \tag{S6}$$

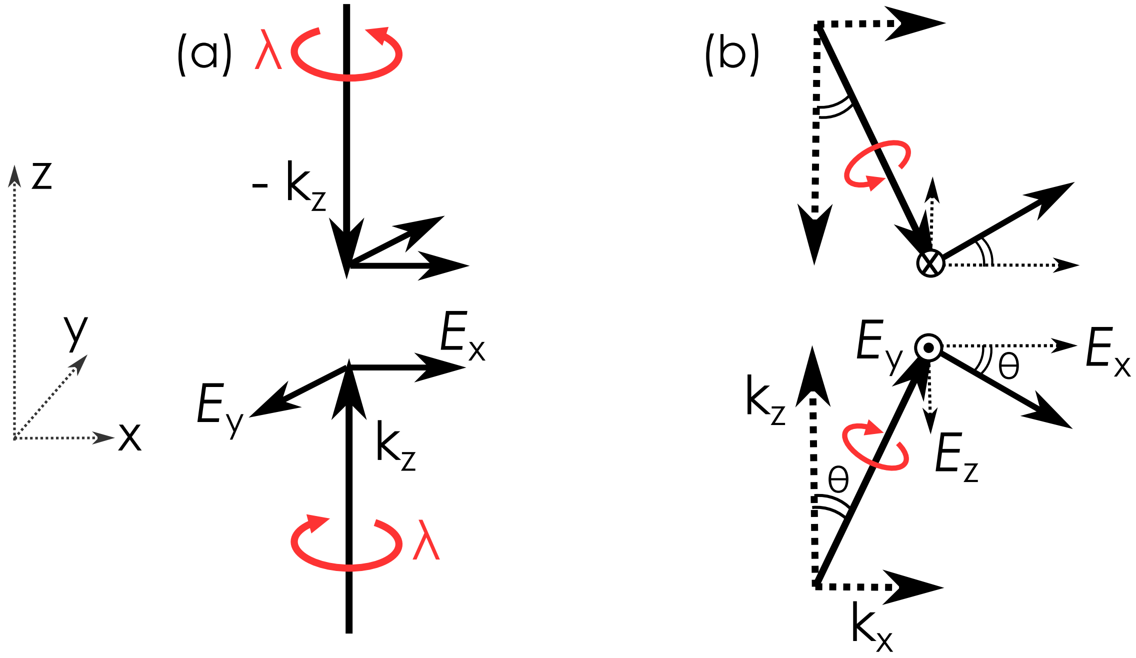

Figure S1: Geometry of a chiral standing wave.

S2 Deriving the chiral Hopfield and Tavis-Cummings models

We start with the chiral standing fields that are derived from the generic mode expansion

$$\hat{\mathbf{D}}_\perp(\mathbf{r}) = i\sum_{\mathbf{k},\lambda}\sqrt{\frac{\hbar ck\varepsilon_0}{2V}}\Big(\epsilon_{\mathbf{k}\lambda}e^{i\mathbf{k}\cdot\mathbf{r}}\hat{a}_{\mathbf{k}\lambda} - \epsilon^*_{\mathbf{k}\lambda}e^{-i\mathbf{k}\cdot\mathbf{r}}\hat{a}^\dagger_{\mathbf{k}\lambda}\Big),$$
$$\hat{\mathbf{B}}(\mathbf{r}) = i\frac{1}{c}\sum_{\mathbf{k},\lambda}\sqrt{\frac{\hbar ck}{2\varepsilon_0 V}}\Big(\beta_{\mathbf{k}\lambda}e^{i\mathbf{k}\cdot\mathbf{r}}\hat{a}_{\mathbf{k}\lambda} - \beta^*_{\mathbf{k}\lambda}e^{-i\mathbf{k}\cdot\mathbf{r}}\hat{a}^\dagger_{\mathbf{k}\lambda}\Big).$$

Following the explanations in the main text, 'upwards' and 'downwards' propagating fields are superimposed as

$$\hat{\mathbf{D}}^\lambda_\perp(\mathbf{r}) = \sum_{\mathbf{k},\lambda}\hat{\mathbf{D}}^\lambda_{\mathbf{k},\perp}(\mathbf{r}),$$
$$\hat{\mathbf{D}}^\lambda_\perp(\mathbf{r}) = \sum_{k}(\hat{\mathbf{D}}^\lambda_{+k,\perp}(\mathbf{r}) + \hat{\mathbf{D}}^\lambda_{-k,\perp}(\mathbf{r}))/\sqrt{2},$$

with $\hat{a}_{+k_z,\lambda} = \hat{a}_{-k_z,\lambda}$ and $\epsilon_{\pm k,\lambda} = \frac{1}{\sqrt{2}}(1, \pm i\lambda, 0)^T$. One obtains

$$\hat{\mathbf{D}}^\lambda_\perp(\mathbf{r}) = -\sum_{k>0}\sqrt{\frac{\varepsilon_0}{V}}\widetilde{\varepsilon}^\lambda_k(z)\hat{p}_{k,\lambda},$$

with $\widetilde{\varepsilon}^\lambda_k(z) = (\cos(kz), -\lambda\sin(kz), 0)^T$ and $\hat{p}_{k,\lambda} = -i\sqrt{\hbar ck/2}(\hat{a}_{\mathbf{k},\lambda} - \hat{a}^\dagger_{\mathbf{k},\lambda})$, $\hat{q}_{k,\lambda} = \sqrt{\hbar/2ck}(\hat{a}_{\mathbf{k},\lambda} + \hat{a}^\dagger_{\mathbf{k},\lambda})$. The same procedure is applied to the magnetic fields where now however $\beta_{\mathbf{k},\lambda} = -i\lambda\epsilon_{\mathbf{k},\lambda}$ such that the chiral standing magnetic field features the additional λ

$$\hat{\mathbf{B}}_\lambda(\mathbf{r}) = \sum_{k>0}\sqrt{k^2/\varepsilon_0 V}\lambda\widetilde{\varepsilon}^\lambda_k(z)\hat{q}_{k,\lambda}.$$

Considering that $\nabla\times\widetilde{\varepsilon}^\lambda_k(z) = \lambda k\widetilde{\varepsilon}^\lambda_k(z)$, it is easily validated that those fields fulfill Maxwell's equations of motion and contribute the common photonic energy $\sum_{k>0}\hbar\omega_k(\hat{a}^\dagger\hat{a} + \frac{1}{2})$ per handedness λ.

We will assume in the following that only a single mode couples substantially to the ensemble of chiral molecules, a reasonable approximation for most cavity realizations that should be however relaxed if the inter-molecular distances become much larger than the wavelength of the electromagnetic standing field. Explicitly expressing all components in the Hamiltonian, as defined in the main text, leads to

$$
\begin{aligned}
\hat{H} &= \sum_n \hat{H}_{M,n} + \frac{1}{2\varepsilon_0 V}\Big[\sum_n \tilde{\varepsilon}_k^\lambda(z)\cdot\hat{\boldsymbol{\mu}}_n\Big]^2 + \frac{1}{2}\hat{p}_k^2 \\
&+ \frac{1}{2}\Big[\omega_k^2 + \sum_{n=1}^{N_{mol}}\sum_{i,j=1}^{3} 2\hat{\chi}^m_{n,ij}\frac{k^2}{\varepsilon_0 V}\tilde{\varepsilon}^\lambda_{k,i}(z)\tilde{\varepsilon}^\lambda_{k,j}(z)\Big]\hat{q}_k^2 \\
&+ \sum_n \sqrt{\frac{1}{\varepsilon_0 V}}\Big[\tilde{\varepsilon}_k^\lambda(z)\cdot\hat{\boldsymbol{\mu}}_n + \sum_{a,b\in\{x,y,z\}}\hat{Q}_{ab,n}\nabla_{a,n}\tilde{\varepsilon}^\lambda_{k,b}(z)\Big]\hat{p}_k \\
&- \sum_n \sqrt{\frac{k^2}{\varepsilon_0 V}}\lambda\tilde{\varepsilon}_k^\lambda(z)\cdot\hat{\mathbf{m}}_n\hat{q}_k.
\end{aligned}
\tag{S7}
$$

The self-magnetization term mediated via $\hat{\chi}^m_{n,ij}$ represents our first obstacle since it combines operators to cubic order, thus going beyond the otherwise quadratic form. In combination with the self-polarization term, the self-magnetization ensures gauge invariance and the stability of the combined system which renders it essential for any future developments of *ab initio* cavity QED. As we strive for a simple analytical solution, we will assume a parametric dependence $\hat{\chi}^m_{n,ij} \approx \chi^m_{n,ij}$ such that $\bar{\omega}_k^2 = \omega_k^2\Big[1+2\sum_n\sum_{i,j=1}^3 \chi^m_{n,ij}\tilde{\varepsilon}^\lambda_{k,i}(z)\tilde{\varepsilon}^\lambda_{k,j}(z)/(c^2\varepsilon_0 V)\Big]$ characterized the effective photonic frequency. While this correction is inherently small ($\propto 1/c^2$), it should be noted that it scales linear in the number of molecules. As $\chi^m_{n,ij}$ is rarely even mentioned and to the best of our knowledge never considered, it remains an open problem to specify its dynamic value. It is however possible to set the value for $\bar{\omega}_k$ in relation to the systems characteristic solution. Using the sum-rule $\langle m|[\hat{p}_k,[\hat{H},\hat{p}_k]]|m\rangle = 2\sum_n(E_n - E_m)|\langle m|\hat{p}_k|n\rangle|^2$ with eigenvalues E_m and eigenstates $|m\rangle$ provides $\bar{\omega}_k^2 = 2\sum_n(E_n - E_m)/\hbar^2|\langle m|\hat{p}_k|n\rangle|^2 \quad \forall\ m$. We will formally retain the self-magnetization term but ultimately ignore its influence on the visualization. It should be noted that the following model features a magnetic instability for sizeable particle number and ξ if $\bar{\omega}_k$ is not adjusted accordingly. We will limit our dis-

cussion to the stable domain, i.e., where phase transitions are absent. Importantly, any *ab initio* calculation that includes the flexibility to change the electric/nuclear structure should include *all* components.[1,2]

S2.1 Transition dipole moments

We will illustrate the identification and relation of the transition moment for the two-level approximation in the following. The corresponding Hopfield model, featuring an identification with harmonic oscillators instead, is derived analogously. We disregard permanent dipole moments for brevity

$$\hat{\boldsymbol{\mu}}_n \rightarrow (\boldsymbol{\mu}_{10}^n \hat{\sigma}_+ + \boldsymbol{\mu}_{01}^n \hat{\sigma}_-), \tag{S8}$$

$$\hat{\mathbf{m}}_n \rightarrow (\mathbf{m}_{10}^n \hat{\sigma}_+ + \mathbf{m}_{01}^n \hat{\sigma}_-), \tag{S9}$$

where the matrix elements of the transition dipole moment (TDM) operators are calculated according to

$$\boldsymbol{\mu}_{01} = \langle 0|\hat{\boldsymbol{\mu}}|1\rangle, \quad \mathbf{m}_{01} = \langle 0|\hat{\mathbf{m}}|1\rangle, \tag{S10}$$

and the lowering and raising operators of the TLS are given by the standard expressions

$$\sigma_+ = |1\rangle\langle 0|, \quad \sigma_- = \sigma_+^\dagger = |0\rangle\langle 1|. \tag{S11}$$

Without the loss of generality, the matrix element of the electric TDM operator may be assumed real-valued, $\boldsymbol{\mu}_{01} = \boldsymbol{\mu}_{01}^*$.

Let us establish the general relationship between transition dipole moments of a two-level quantum emitter. For a bi-isotropic molecule with parallel electric and magnetic transition dipole moments this equation takes the simple form:[3]

$$\mathbf{m}_{01}^n = -ic\xi \boldsymbol{\mu}_{01}^n, \tag{S12}$$

with $\xi = \pm 1$ corresponding to LH (+1) and RH (−1) emitters, respectively. Correspondingly, the magnetic dipole moment operator becomes $\hat{\mathbf{m}} = ic\xi(\boldsymbol{\mu}_{01}^{*}\hat{\sigma}_{+} - \boldsymbol{\mu}_{01}\hat{\sigma}_{-})$. The above relationship between the TDMs of a chiral emitter is consistent with the chirality definition of a classical monochromatic dipolar source.[4] Let us now look for a more general tensorial expression that would relate the transition dipole moments of an anisotropic molecular emitter.

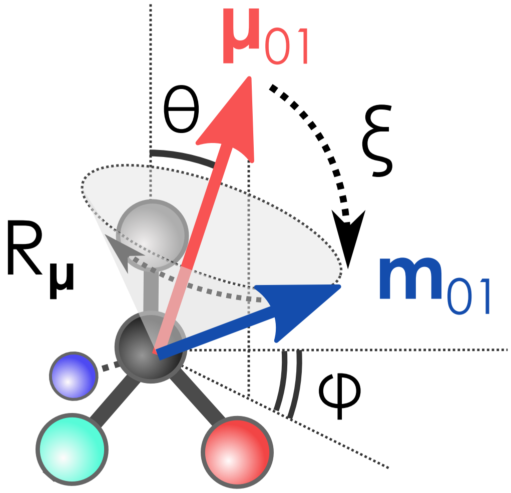

Figure S2: Illustration of the relationship between the electric and magnetic transition dipole moments in a generic molecular emitter. The orientation of the transition electric dipole moment $\boldsymbol{\mu}_{01}$ associated with the molecule can be described by two angles θ, φ in a local spherical coordinates system. $\overset{\leftrightarrow}{\xi}$ describes a unitary mapping $\boldsymbol{\mu} \to \mathbf{m}$. For a given orientation of the electric dipole moment $\boldsymbol{\mu}_{01}$, all allowed positions of $\mathbf{m}_{01}$ occupy a circle denoted by the shaded area. This additional mapping is accomplished by the rotation of the molecule around $\boldsymbol{\mu}_{01}$, which is described by R_{μ}.

Three Euler angles unambiguously describe the orientation of any rigid body (such as a molecule) in space. Similarly, a pair of non-collinear vectors $\boldsymbol{\mu}_{01}$ and $\mathbf{m}_{01}$ hard-wired to the molecule would work. But $\boldsymbol{\mu}_{01}$ and $\mathbf{m}_{01}$ are not independent themselves, and must satisfy a molecule-specific characteristic equation. First let us fix the orientation of the electric dipole moment of the molecule $\boldsymbol{\mu}_{01}$. This vector needs to be mapped into the magnetic transition dipole moment $\mathbf{m}_{01}$. Suppose this mapping is performed by a dyad $\overset{\leftrightarrow}{\xi}$, as illustrated in Fig. S2. This dyad cannot be an arbitrary linear transformation: the action of $\overset{\leftrightarrow}{\xi}$ on $\boldsymbol{\mu}_{01}$ must be invariant with respect to an arbitrary rotation of the molecule. Thus, $\overset{\leftrightarrow}{\xi}$ must be a product

of an orthogonal linear transformation $\overleftrightarrow{U}$ in $\mathbb{R}^3$ and scaling by a complex number s:

$$\overleftrightarrow{\xi} = s \cdot \overleftrightarrow{U}. \tag{S13}$$

We can further specify its form by incorporating two rotations into the spatial orientation of $\boldsymbol{\mu}_{01}$. Choosing a linearly-polarized transition electric dipole moment $\boldsymbol{\mu}_{01}$ is equivalent to fixing two Euler angles of the molecular orientation, leaving an arbitrary rotation around $\boldsymbol{\mu}_{01}$. For example, we can parameterize the electric TDM by the polar and azimuthal angles in the spherical coordinate system:

$$\boldsymbol{\mu}_{01} = |\boldsymbol{\mu}| \begin{pmatrix} \sin\theta\cos\varphi \\ \sin\theta\sin\varphi \\ \cos\theta \end{pmatrix}. \tag{S14}$$

A given tensor $\overleftrightarrow{\xi}$ will map a given electric dipole moment $\boldsymbol{\mu}_{01}$ into another fixed vector. However, given a fixed $\boldsymbol{\mu}_{01}$ all allowed positions of $\mathbf{m}_{01}$ occupy an entire circle in $\mathbb{R}^3$. Thus, mapping $\boldsymbol{\mu}_{01} \to \mathbf{m}_{01}$ must be parameterized by an additional angle describing rotations of the molecule around $\boldsymbol{\mu}_{01}$ (see Fig. S2). The sought for mapping thus can be written as:

$$\mathbf{m}_{01} = -ic\overleftrightarrow{R}_{\mu}(\delta)\overleftrightarrow{\xi}\boldsymbol{\mu}_{01}, \tag{S15}$$

where $\overleftrightarrow{R}_{\mu}(\delta)$ is the rotation matrix that describes rotation of the molecule around $\boldsymbol{\mu}_{01}$ by an angle δ. The mapping parameterized by three angles $(\theta, \varphi, \delta)$ in Eq. S15 encompasses all possible orientations of the molecule, which allows us to average any characteristic of the coupled system (such as the coupling constant) over molecular orientation.

Let us now establish the constraints imposed on $\overleftrightarrow{\xi}$ by reciprocity. The linear relationship between induced electric and magnetic dipole moments of a polarizable subwavelength object

and incident monochromatic field can be written as:

$$\begin{pmatrix} \mathbf{p} \\ \mathbf{m} \end{pmatrix} = \begin{pmatrix} \varepsilon_0 \overleftrightarrow{\alpha}_e & \overleftrightarrow{\alpha}_{em}/c \\ \varepsilon_0 c \overleftrightarrow{\alpha}_{me} & \overleftrightarrow{\alpha}_m \end{pmatrix} \begin{pmatrix} \mathbf{E} \\ \mathbf{H} \end{pmatrix}, \tag{S16}$$

where $\overleftrightarrow{\varepsilon}$, $\overleftrightarrow{\mu}$, $\overleftrightarrow{\alpha}_{em}$, and $\overleftrightarrow{\alpha}_{me}$ are all rank-2 3×3 tensors with units of volume.

We limit our treatment to the class of reciprocal media; polarizabilities of any reciprocal particle are subject to Onsager-Casimir relations:[5]

$$\begin{aligned} \overleftrightarrow{\alpha}_e &= \overleftrightarrow{\alpha}_e^T, \\ \overleftrightarrow{\alpha}_m &= \overleftrightarrow{\alpha}_m^T, \\ \overleftrightarrow{\alpha}_{em} &= -\overleftrightarrow{\alpha}_{me}^T. \end{aligned} \tag{S17}$$

This criterion allows us to decompose the magneto-electric coupling tensors into reciprocal ('R') and non-reciprocal ('NR') components:

$$\overleftrightarrow{\alpha}_{em} = \overleftrightarrow{\alpha}_{em}^{(NR)} + \overleftrightarrow{\alpha}_{em}^{(R)} \equiv \overleftrightarrow{\chi} + i\overleftrightarrow{\kappa}, \tag{S18}$$

$$\overleftrightarrow{\alpha}_{me} = \overleftrightarrow{\alpha}_{me}^{(NR)} + \overleftrightarrow{\alpha}_{me}^{(R)} \equiv \overleftrightarrow{\chi}^T - i\overleftrightarrow{\kappa}^T. \tag{S19}$$

where the reciprocal part is presented by $\overleftrightarrow{\kappa}$:

$$\kappa = \frac{\overleftrightarrow{\alpha}_{em} - \overleftrightarrow{\alpha}_{me}^T}{2i}, \tag{S20}$$

and the non-reciprocal part is presented by

$$\chi = \frac{\overleftrightarrow{\alpha}_{em} + \overleftrightarrow{\alpha}_{me}^T}{2}. \tag{S21}$$

Obviously, if $\overleftrightarrow{\chi} = 0$, then $\overleftrightarrow{\alpha}_{em} = -\overleftrightarrow{\alpha}_{me}^T$, and the reciprocity criterion is satisfied. It is self-explanatory that the reciprocal part of magneto-electric polarizability is responsible for

effects that respect reciprocity.

Atomic polarizabilities of the elementary two-level system with arbitrary transition dipole moments $\boldsymbol{\mu}$ and $\mathbf{m}$ can be written as:

$$\overleftrightarrow{\alpha}_e = \frac{\boldsymbol{\mu}_{01} \otimes \boldsymbol{\mu}_{01}^*}{\hbar\varepsilon_0} \frac{1}{\omega_0 - \omega - i\gamma/2}, \quad \overleftrightarrow{\alpha}_m = \frac{\mathbf{m}_{01} \otimes \mathbf{m}_{01}^*}{\hbar\varepsilon_0 c^2} \frac{1}{\omega_0 - \omega - i\gamma/2}, \tag{S22}$$

$$\overleftrightarrow{\alpha}_{em} = \frac{\boldsymbol{\mu}_{01} \otimes \mathbf{m}_{01}^*}{\hbar\varepsilon_0 c} \frac{1}{\omega_0 - \omega - i\gamma/2}, \quad \overleftrightarrow{\alpha}_{me} = \frac{\mathbf{m}_{01} \otimes \boldsymbol{\mu}_{01}^*}{\hbar\varepsilon_0 c} \frac{1}{\omega_0 - \omega - i\gamma/2}. \tag{S23}$$

Equations S22 suggest that for $\overleftrightarrow{\alpha}_e$ and $\overleftrightarrow{\alpha}_m$ to comply with Casimir-Onsager relations, Eq. S17, $\boldsymbol{\mu}_{01}$ and $\mathbf{m}_{01}$ must be real-valued vectors (up to an arbitrary global phase $e^{i\phi}$), thus describing linearly-polarized transitions. One can easily see that plugging $\mathbf{m}_{01} = -ic\xi\boldsymbol{\mu}_{01}$ (Eq. S12) into the above expressions yields $\overleftrightarrow{\alpha}_{em} = -\overleftrightarrow{\alpha}_{me}^T$ and $\overleftrightarrow{\chi} = 0$.

Now let us utilize Eq. S15 and for brevity work with the numerator of the full expression in Eq. S23:

$$\begin{aligned} \overleftrightarrow{\alpha}_{em} \propto \boldsymbol{\mu}_{01} \otimes \mathbf{m}_{01}^* \equiv \boldsymbol{\mu}_{01}\mathbf{m}_{01}^\dagger = \boldsymbol{\mu}_{01}\left(-ic\overleftrightarrow{R}_{\boldsymbol{\mu}}\overleftrightarrow{\xi}\boldsymbol{\mu}_{01}\right)^\dagger \\ = \boldsymbol{\mu}_{01}\left(-ic\boldsymbol{\mu}_{01}^T(\overleftrightarrow{R}_{\boldsymbol{\mu}}\overleftrightarrow{\xi})^T\right)^* = ic\boldsymbol{\mu}_{01}\boldsymbol{\mu}_{01}^\dagger(\overleftrightarrow{R}_{\boldsymbol{\mu}}\overleftrightarrow{\xi})^\dagger. \end{aligned} \tag{S24}$$

Similarly, for α_{me} we obtain:

$$\overleftrightarrow{\alpha}_{me} \propto \mathbf{m}_{01} \otimes \boldsymbol{\mu}_{01}^* \equiv \mathbf{m}_{01}\boldsymbol{\mu}_{01}^\dagger = -ic\left(\overleftrightarrow{R}_{\boldsymbol{\mu}}\overleftrightarrow{\xi}\right)\boldsymbol{\mu}_{01}\boldsymbol{\mu}_{01}^\dagger. \tag{S25}$$

Transposing the latter, assuming without the loss of generality the real-valued $\boldsymbol{\mu}_{01}$ and inserting into the Casimir-Onsager relation, we get:

$$\overleftrightarrow{\alpha}_{em} + \overleftrightarrow{\alpha}_{me}^T \propto ic\boldsymbol{\mu}_{01}\boldsymbol{\mu}_{01}^\dagger[(\overleftrightarrow{R}_{\boldsymbol{\mu}}\overleftrightarrow{\xi})^\dagger - (\overleftrightarrow{R}_{\boldsymbol{\mu}}\overleftrightarrow{\xi})^T] = 0. \tag{S26}$$

Since $\overleftrightarrow{R}_{\boldsymbol{\mu}}$ is a real-valued orthogonal matrix, the latter implies that for a reciprocal bi-

anisotropic two-level emitter $\overleftrightarrow{\xi}$ must be real-valued:

$$\boxed{\mathfrak{Im}[\overleftrightarrow{\xi}] = 0.} \tag{S27}$$

In other words, the scaling factor s of the transformation is real-valued, $\mathfrak{Im}[s] = 0$.

For now, let us assume that the transition dipole moments are related by the simple expression with a scalar ξ, Eq. S12. Using this compact relation between electric and magnetic moments allows us to combine electric dipole, electric quadrupole and magnetic dipole into a single compact expression (assuming $\boldsymbol{\mu}_{10}^{n} = \boldsymbol{\mu}_{10}^{n,*}$ and $Q_{ab,n}^{10} = Q_{ab,n}^{01}$, $\mathbf{Q}_n = Q_{ab,n}^{10}\nabla_a \mathbf{e}_b$):

$$\begin{aligned} \hat{H} = &\sum_n \hat{H}_{M,n} + \frac{1}{2\varepsilon_0 V}\Big[\sum_n \tilde{\varepsilon}_k^{\lambda}(z)\cdot\boldsymbol{\mu}_{01}^{n}(\sigma_n^+ + \sigma_n^-)\Big]^2 + \hbar\bar{\omega}_k\left(\hat{a}^\dagger\hat{a} + \frac{1}{2}\right) \\ &- i\sum_n \bar{g}_n\Big[(\sigma_n^+ + \sigma_n^-)(\hat{a} - \hat{a}^\dagger) - \bar{\xi}_n\lambda(\sigma_n^+ - \sigma_n^-)(\hat{a} + \hat{a}^\dagger)\Big] \end{aligned} \tag{S28}$$

where

$$\bar{g}_n = \sqrt{\frac{\hbar\bar{\omega}_k}{2\varepsilon_0 V}}(\boldsymbol{\mu}_{10}^{n} + \mathbf{Q}_n)\cdot\tilde{\varepsilon}_k^{\lambda}(z) \tag{S29}$$

and

$$\bar{\xi}_n = \frac{\omega_k}{\bar{\omega}_k}\frac{\boldsymbol{\mu}_{10}^{n}\cdot\tilde{\varepsilon}_k^{\lambda}(z)}{(\boldsymbol{\mu}_{10}^{n} + \mathbf{Q}_n)\cdot\tilde{\varepsilon}_k^{\lambda}(z)}\xi. \tag{S30}$$

S2.2 Tavis-Cummings models – a hint at the effective coupling strength

Let us here introduce the common matter-representation of two-level models, discard the self-polarization term and all counter-rotating terms ($\propto \hat{a}\hat{\sigma}^- \approx 0$ and similar). We obtain the strongly simplified chiral Tavis-Cummings Hamiltonian:

$$\hat{H}_{CTC} = \sum_n \hbar\omega_m\hat{\sigma}_n^+\hat{\sigma}_n^- + \hbar\bar{\omega}_k(\hat{a}^\dagger\hat{a} + \frac{1}{2}) - i\hbar\sum_n \bar{g}_n(1 + \bar{\xi}_n\lambda)\Big[\hat{\sigma}_n^+\hat{a} - \hat{\sigma}_n^-\hat{a}^\dagger\Big] \tag{S31}$$

that shows explicitly that the effective interaction strength is proportional to $\bar{g}_n(1+\bar{\xi}_n\lambda)$. A chiral emitter that features the same handedness as the cavity will couple stronger to the mode. In the extreme case that $\bar{\xi}_n = \pm 1$, the mismatched enantiomer will entirely decouple from the mode. The above chiral Tavis-Cummings model could be solved analytically in the same way as any Tavis-Cummings model, i.e, by limiting ourselves to the single-excitation subspace and introducing collective spin-operators. Here, we will focus on the Hopfield model that includes also the counter-rotating and self-polarization terms that can lead to sizeable renormalizations for large N.

S2.3 Chiral Hopfield model

In contrast to the Dicke and Tavis-Cummings models with a two-level approximation, the Hopfield approach is based on representing the material in terms of harmonic oscillators which allows for an analytic solution also in the ultra-strong coupling domain. The first polariton manifold is identical in the single-excitation + strong-coupling regime. Our Chiral Hopfield Hamiltonian takes the form of $N+1$ coupled harmonic oscillators

$$\begin{aligned}\hat{H} &= \sum_n \hbar\omega_n(\hat{b}_n^\dagger\hat{b}_n + \frac{1}{2}) + \hbar\bar{\omega}_k(\hat{a}^\dagger\hat{a} + \frac{1}{2}) \\ &- i\hbar\sum_n \bar{g}_n\Big[(\hat{b}_n^\dagger + \hat{b}_n)(\hat{a} - \hat{a}^\dagger) + \bar{\xi}_n\lambda(\hat{b}_n^\dagger - \hat{b}_n)(\hat{a} + \hat{a}^\dagger)\Big] \\ &+ \frac{1}{2\varepsilon_0 V}\Big[\sum_n \tilde{\varepsilon}_k^\lambda(z)\cdot\boldsymbol{\mu}_{01}^n(\hat{b}_n^\dagger + \hat{b}_n)\Big]^2.\end{aligned} \tag{S32}$$

The transition-dipole moments are related to the optical oscillator strength of the harmonic model. Assuming identical molecules, it is convenient to introduce the Fourier-representation for the molecular ensemble

$$\hat{B}_{\mathbf{k}}^\dagger = \frac{1}{\sqrt{N}}\sum_n e^{i\mathbf{k}\cdot\mathbf{r}_n}\hat{b}_n^\dagger,\ \ \hat{b}_n^\dagger = \frac{1}{\sqrt{N}}\sum_{\mathbf{k}} e^{-i\mathbf{k}\cdot\mathbf{r}_n}\hat{B}_{\mathbf{k}}^\dagger \tag{S33}$$

such that the collective operators are represented by bright $\sum_n \hat{b}^\dagger = \sqrt{N}\hat{B}^\dagger_{k=0}$ and dark states

$$\begin{aligned}\hat{H} \approx\ & \hbar\omega_m(\hat{B}^\dagger_{k=0}\hat{B}_{k=0} + \frac{1}{2}) + \hbar\omega_m \sum_{k\neq 0} \hat{B}^\dagger_k \hat{B}_k + (N-1)\frac{\hbar\omega_m}{2} + \hbar\bar{\omega}_k(\hat{a}^\dagger\hat{a} + \frac{1}{2}) \\ & - i\hbar\sqrt{N}\bar{g}\Big[(\hat{B}^\dagger_{k=0} + \hat{B}_{k=0})(\hat{a} - \hat{a}^\dagger) + \bar{\xi}\lambda(\hat{B}^\dagger_{k=0} - \hat{B}_{k=0})(\hat{a} + \hat{a}^\dagger)\Big] \\ & + \frac{N}{2\varepsilon_0 V}\Big[\widetilde{\varepsilon}^\lambda_k(z) \cdot \boldsymbol{\mu}_{01}(\hat{B}^\dagger_{k=0} + \hat{B}_{k=0})\Big]^2 \ .\end{aligned} \tag{S34}$$

Disregarding the dark states $\hbar\omega_m \sum_{k\neq 0} \hat{B}^\dagger_k \hat{B}_k$ and their vacuum-fluctuations $(N-1)\frac{\hbar\omega_m}{2}$, the self-polarization term can be absorbed into an adjusted matter frequency of the bright states $\widetilde{\omega}^2_m = \omega^2_m + N\frac{2\omega_m}{\hbar\varepsilon_0 V}(\widetilde{\varepsilon}^\lambda_k(z)\cdot\boldsymbol{\mu}_{01})^2$ which results in

$$\begin{aligned}\hat{H} =\ & \hbar\widetilde{\omega}_m(\hat{B}^\dagger_{k=0}\hat{B}_{k=0} + \frac{1}{2}) + \hbar\bar{\omega}_k(\hat{a}^\dagger\hat{a} + \frac{1}{2}) \\ & - i\hbar\sqrt{N}\widetilde{g}\Big[(\hat{B}^\dagger_{k=0} + \hat{B}_{k=0})(\hat{a} - \hat{a}^\dagger) + \widetilde{\xi}\lambda(\hat{B}^\dagger_{k=0} - \hat{B}_{k=0})(\hat{a} + \hat{a}^\dagger)\Big] \ .\end{aligned} \tag{S35}$$

where $\widetilde{g} = \sqrt{\frac{\hbar\bar{\omega}_k\omega_m}{2\varepsilon_0 V\widetilde{\omega}_m}}(\boldsymbol{\mu}_{01}+\mathbf{Q}_n)\cdot\widetilde{\varepsilon}^\lambda_k(z)$ and $\widetilde{\xi} = \frac{\widetilde{\omega}_m\omega_k}{\omega_m\bar{\omega}_k}\frac{\boldsymbol{\mu}_{01}\cdot\widetilde{\varepsilon}^\lambda_k(z)}{(\boldsymbol{\mu}_{01}+\mathbf{Q})\cdot\widetilde{\varepsilon}^\lambda_k(z)}\xi$ are the renormalized effective coupling strength and chirality factor.

We can diagonalize Eq. (S35) by following the standard Hopfield[6,7] procedure, i.e., defining the polaritonic operator $\hat{\Pi} = x\hat{a} + y\hat{a}^\dagger + z\hat{B}_{k=0} + u\hat{B}^\dagger_{k=0}$ that fulfills the eigenvalue equation $[\hat{H}, \hat{\Pi}] = \hbar\Omega\hat{\Pi}$ with the normalization condition $|x|^2 - |y|^2 + |z|^2 - |u|^2 = 1$. We obtain the polaritonic frequencies from

$$\begin{vmatrix} -\bar{\omega}_k - \Omega & 0 & (1+\widetilde{\xi}\lambda)i\sqrt{N}\widetilde{g} & -(1-\widetilde{\xi}\lambda)i\sqrt{N}\widetilde{g} \\ 0 & \bar{\omega}_k - \Omega & -(1-\widetilde{\xi}\lambda)i\sqrt{N}\widetilde{g} & (1+\widetilde{\xi}\lambda)i\sqrt{N}\widetilde{g} \\ -(1+\widetilde{\xi}\lambda)i\sqrt{N}\widetilde{g} & -(1-\widetilde{\xi}\lambda)i\sqrt{N}\widetilde{g} & -\widetilde{\omega}_m - \Omega & 0 \\ -(1-\widetilde{\xi}\lambda)i\sqrt{N}\widetilde{g} & -(1+\widetilde{\xi}\lambda)i\sqrt{N}\widetilde{g} & 0 & \widetilde{\omega}_m - \Omega \end{vmatrix} = 0 \tag{S36}$$

as real and positive solutions

$$\Omega_\pm = \frac{1}{\sqrt{2}}\sqrt{\bar{\omega}^2_k + \widetilde{\omega}^2_m + 8\widetilde{\xi}\lambda N\widetilde{g}^2 \pm \sqrt{[\omega^2_k - \widetilde{\omega}^2_m]^2 + 16N\widetilde{g}^2(\bar{\omega}_k + \widetilde{\omega}_m\widetilde{\xi}\lambda)(\bar{\omega}_k\widetilde{\xi}\lambda + \widetilde{\omega}_m)}} \ . \tag{S37}$$

The corresponding eigenvectors encode in $|x|^2-|y|^2$ the matter contribution and in $|z|^2-|u|^2$ the photonic contribution to the polaritonic states.

S2.4 Generic alignment and its influence on chiral recognition

Let us briefly examine the more general case where the electric and magnetic transition dipole moments are arbitrarily oriented. In this case, they are related by Eq. S22 with $\overset{\leftrightarrow}{\xi} = s\overset{\leftrightarrow}{U}$ where s is real-valued and $\overset{\leftrightarrow}{U}$ is an orthogonal transformation.

The orientation average is described by the energy-conserving squared coupling element:

$$\langle |g|^2 \rangle \propto \frac{1}{4\pi}\int_0^{2\pi} d\phi \int_0^{\pi} d\theta \sin(\theta) |\sqrt{N}\tilde{\varepsilon}\cdot(1+\lambda\overset{\leftrightarrow}{\xi})\boldsymbol{\mu}_{01}|^2 \tag{S38}$$

proportional to the combined electric plus magnetic moments. Expanding the dot product

$$\begin{aligned} |\tilde{\varepsilon}\cdot(1+\lambda\overset{\leftrightarrow}{\xi})\boldsymbol{\mu}_{01}|^2 &= \cos^2\theta |(1+\lambda\overset{\leftrightarrow}{\xi})\boldsymbol{\mu}_{01}|^2 \\ &= \cos^2\theta\left(|\boldsymbol{\mu}_{01}|^2 + \langle \overset{\leftrightarrow}{\xi}\boldsymbol{\mu}_{01}|\overset{\leftrightarrow}{\xi}\boldsymbol{\mu}_{01}\rangle + 2\lambda\langle\boldsymbol{\mu}_{01}|\overset{\leftrightarrow}{\xi}\boldsymbol{\mu}_{01}\rangle\right) \end{aligned} \tag{S39}$$

and utilizing $\overset{\leftrightarrow}{\xi} = s\overset{\leftrightarrow}{U}$, we obtain:

$$\begin{aligned} \langle |g|^2\rangle &= |\sqrt{\frac{\hbar\bar{\omega}_k\omega_m}{2\varepsilon_0 V\tilde{\omega}_m}}\frac{\tilde{\omega}_m\omega_k}{\omega_m\bar{\omega}_k}\sqrt{N}(1+\lambda\overset{\leftrightarrow}{\xi})\cdot\boldsymbol{\mu}_{01}|^2\frac{1}{2}\int_0^{\pi} d\theta\sin(\theta)\cos^2(\theta) \\ &= \frac{N}{3}\frac{\hbar\tilde{\omega}_m\omega_k^2}{2\varepsilon_0 V\bar{\omega}_k\omega_m}|(1+\lambda\overset{\leftrightarrow}{\xi})\cdot\boldsymbol{\mu}_{01}|^2 \\ &= \frac{N}{3}\frac{\hbar\tilde{\omega}_m\omega_k^2}{2\varepsilon_0 V\bar{\omega}_k\omega_m}\left[(1+s^2)|\boldsymbol{\mu}_{01}|^2 + 2\lambda s\Re\mathrm{e}\langle\boldsymbol{\mu}_{01}|\overset{\leftrightarrow}{U}\boldsymbol{\mu}_{01}\rangle\right]. \end{aligned} \tag{S40}$$

In addition to chiral features that arise from the parallel components of the transition dipole moments, the emitters now also feature Omega-type magneto-electric coupling originating from the orthogonal components of the dipole moments. However, as we can easily see from the angular average, only the chiral components are discriminated by the cavity. Eq. (S40) clarifies that the chiral cavity will only distinguish the chiral components of the emitters. Take for example pure chirality with $\lambda = +1$ and (anti-)alignment $\boldsymbol{\mu}||\mathbf{m}$ with

$\overleftrightarrow{\xi} = \pm 1$, then $[(1+s^2)|\boldsymbol{\mu}_{01}|^2 + 2\lambda s \Re\mathrm{e}\langle \boldsymbol{\mu}_{01}|\overleftrightarrow{U}_\xi \boldsymbol{\mu}_{01}\rangle] = (1+1+2(\pm 1))|\boldsymbol{\mu}_{01}|^2$, which is either 0 or $4|\boldsymbol{\mu}_{01}|^2$. However, for Ω-coupling $\langle \boldsymbol{\mu}_{01}|\overleftrightarrow{U}\boldsymbol{\mu}_{01}\rangle = 0$ and we obtain always $(1+s^2)|\boldsymbol{\mu}_{01}|^2$. The transition-dipole moments still contribute constructively to the chiral coupling but there is no handedness selectivity left.

S2.5 Influence of the self-magnetization

Let us illustrate briefly how the self-magnetization can influence our conclusions. First of all, it should be noticed that the dressing of the photonic frequency via χ^m is a factor $1/c^2$ smaller than the self-polarization effect on the matter frequency. However, even if we chose enormously large values, the effect is small as demonstrated in Fig. S3.

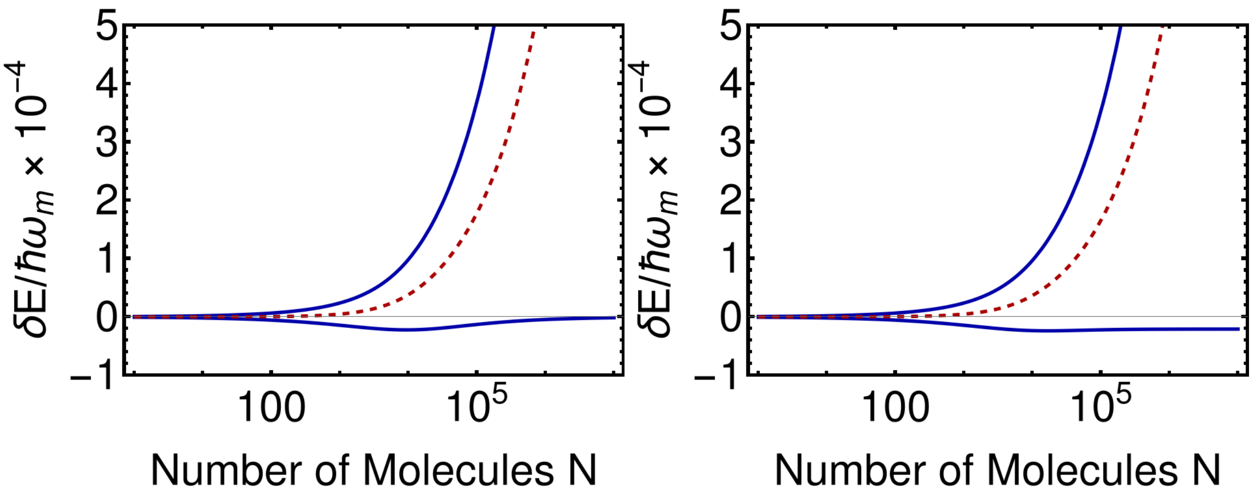

Figure S3: Left, including a quite large self-magnetization of $\chi^m = \mu^2$. Right, using a vastly enhanced self-magnetization of $\chi^m = c^2\mu^2$, so even if we compensate the $1/c^2$ factor, the effect of the self-magnetization is small. Interestingly, the large self-magnetization can even enhance the recognition capabilities in the lower polariton.

S2.6 Extension to modes with non-zero in-plane momentum $k_x \neq 0$

Our previous derivations used the simplifying assumption of a cavity mode represented by a standing wave with $\mathbf{k} = \pm k\mathbf{e}_z$, which leads to compact and highly intuitive equations.

A more generic description might allow for non-zero in-plane momentum $k_x \neq 0$, resulting in mixing of propagating waves and dark states. We will provide in the following a brief discussion what such an extension would look like and what changes are to be expected. We would like to emphasize that such an extended model would go more naturally with a many-mode description and present a straightforward generalization of our work.

A planar optical cavity, such as the one described in ref.,[8] supports a continuous spectrum of resonant states that can be labeled by their in-plane momenta $\mathbf{k}_{\parallel}$. More sophisticated cavities, such as micro-domes, support more complex modes with in-plane contribution and non-Gaussian spot-distribution but we retain here with the simplified Fabry-Pérot set up. A minimal representation for the field of such cavity modes is given in Section *Fields of a standing chiral wave.* Cavity modes with $\mathbf{k}_{\parallel} \neq \mathbf{0}$ do maintain their single-handedness quality in a substantial range of in-plane wave vectors (incident angles) according to the findings of ref.,[8] and thus are expected to feature similar energy spectra when coupled with chiral molecular emitters.

As before, we can expand the fields in its eigenmodes

$$\hat{\mathbf{D}}_{\perp}^{\lambda}(\mathbf{r}) = i \sum_{\mathbf{k}} \sqrt{\frac{\hbar c k \varepsilon_0}{2V}} \Big(\epsilon_{\mathbf{k}\lambda}(\mathbf{r}) \hat{a}_{\mathbf{k}\lambda} - \epsilon_{\mathbf{k}\lambda}^{*}(\mathbf{r}) \hat{a}_{\mathbf{k}\lambda}^{\dagger} \Big),$$

$$\hat{\mathbf{B}}^{\lambda}(\mathbf{r}) = i \frac{1}{c} \sum_{\mathbf{k}} \sqrt{\frac{\hbar c k}{2\varepsilon_0 V}} \Big(\beta_{\mathbf{k}\lambda}(\mathbf{r}) \hat{a}_{\mathbf{k}\lambda} - \beta_{\mathbf{k}\lambda}^{*}(\mathbf{r}) \hat{a}_{\mathbf{k}\lambda}^{\dagger} \Big)$$

where $\epsilon_{\mathbf{k}\lambda}(\mathbf{r}) = \frac{1}{\sqrt{2}}(k_z/k, i\lambda, -k_x/k)^T e^{ik_z z + ik_x x}$ and $\beta_{\mathbf{k}\lambda}(\mathbf{r}) = -i\lambda \epsilon_{\mathbf{k}\lambda}(\mathbf{r})$ such that

$$\hat{\mathbf{B}}^{\lambda}(\mathbf{r}) = \frac{1}{c} \sum_{\mathbf{k}} \lambda \sqrt{\frac{\hbar c k}{2\varepsilon_0 V}} \Big(\epsilon_{\mathbf{k}\lambda}(\mathbf{r}) \hat{a}_{\mathbf{k}\lambda} + \epsilon_{\mathbf{k}\lambda}^{*}(\mathbf{r}) \hat{a}_{\mathbf{k}\lambda}^{\dagger} \Big).$$

We again restrict enforce the chiral standing wave in z-direction by $\hat{\mathbf{D}}_{\perp}^{\lambda}(\mathbf{r}) = \frac{1}{\sqrt{2}}(\hat{\mathbf{D}}_{\perp,k_z>0}^{\lambda}(\mathbf{r}) +$

$\hat{\mathbf{D}}^{\lambda}_{\perp,k_z<0}(\mathbf{r}))$ which results with $\hat{a}_{k_x,k_z>0,\lambda} = \hat{a}_{k_x,k_z<0,\lambda}$ in

$$\hat{\mathbf{D}}^{\lambda}_{\perp}(\mathbf{r}) = i \sum_{k_z>0,k_x} \sqrt{\frac{\hbar c k \varepsilon_0}{2V}} \Big(\widetilde{\epsilon}_{\mathbf{k}\lambda}(\mathbf{r})\hat{a}_{\mathbf{k}\lambda} - \widetilde{\epsilon}^{*}_{\mathbf{k}\lambda}(\mathbf{r})\hat{a}^{\dagger}_{\mathbf{k}\lambda}\Big),$$
$$\hat{\mathbf{B}}^{\lambda}(\mathbf{r}) = \frac{1}{c} \sum_{k_z>0,k_x} \lambda \sqrt{\frac{\hbar c k}{2\varepsilon_0 V}} \Big(\widetilde{\epsilon}_{\mathbf{k}\lambda}(\mathbf{r})\hat{a}_{\mathbf{k}\lambda} + \widetilde{\epsilon}^{*}_{\mathbf{k}\lambda}(\mathbf{r})\hat{a}^{\dagger}_{\mathbf{k}\lambda}\Big)$$

where $\widetilde{\epsilon}_{\mathbf{k}\lambda}(\mathbf{r}) = (k_z/k\cos(k_z z), -\lambda\sin(k_z z), -ik_x/k\sin(k_z z))^T e^{ik_x x}$.

The projection of the matter degrees of freedom follows as before and the overall structure remains unchanged. As an example, we will derive the new Tavis-Cummings analogue, the Hopfield model can be obtain in analogy to the previous steps but the many-mode coupling renders the process more verbose. As before, we perform the rotating-wave approximation for the chiral Tavis-Cummings model and disregard all self-correction terms as well as quadrupole contributions to obtain

$$\begin{aligned}
\hat{H}^{\lambda}_{CTC} &= \sum_n \hbar\omega_m \hat{\sigma}^{+}_n \hat{\sigma}^{-}_n + \sum_{k_z>0,k_x} \hbar\omega_{\mathbf{k}} (\hat{a}^{\dagger}_{\mathbf{k}\lambda}\hat{a}_{\mathbf{k}\lambda} + \frac{1}{2}) \\
&- \sum_{n,k_z>0,k_x} i\sqrt{\frac{\hbar c k}{2\varepsilon_0 V}} \Big[(\widetilde{\epsilon}_{\mathbf{k}\lambda}(\mathbf{r}) \cdot \boldsymbol{\mu}^{n}_{01} \hat{\sigma}^{+}_n \hat{a}_{\mathbf{k}\lambda} - h.a.) + \lambda(\widetilde{\epsilon}_{\mathbf{k}\lambda}(\mathbf{r}) \overleftrightarrow{\xi}_n \boldsymbol{\mu}^{n}_{01} \hat{\sigma}^{+}_n \hat{a}_{\mathbf{k}\lambda} - h.a.)\Big]. \\
&= \sum_n \hbar\omega_m \hat{\sigma}^{+}_n \hat{\sigma}^{-}_n + \sum_{k_z>0,k_x} \hbar\omega_{\mathbf{k}} (\hat{a}^{\dagger}_{\mathbf{k}\lambda}\hat{a}_{\mathbf{k}\lambda} + \frac{1}{2}) - \sum_{n,k_z>0,k_x} i\sqrt{\frac{\hbar c k}{2\varepsilon_0 V}} \Big[\widetilde{\epsilon}_{\mathbf{k}\lambda}(\mathbf{r})(1+\lambda\overleftrightarrow{\xi}_n) \cdot \boldsymbol{\mu}^{n}_{01} \hat{\sigma}^{+}_n \hat{a}_{\mathbf{k}\lambda} - h.a.\Big].
\end{aligned}$$

We introduce again a Fourier-representation $\hat{\sigma}^{+}_n = \frac{1}{\sqrt{N}} \sum_{\mathbf{K}} e^{-i\mathbf{K}\cdot\mathbf{r}_n} \hat{S}^{\dagger}_{\mathbf{K}}$ which implies a regular molecular distance along the x-axis $\mathbf{r}_n = \mathbf{e}_x \frac{2\pi n}{N}$ and assume identical couplings and frequencies. As $\sum_n e^{ik_x x_n} e^{-iK_x x_n} = N\delta(k_x - K_x)$, the chiral Tavis-Cummings Hamiltonian simplifies to

$$\begin{aligned}
\hat{H}^{\lambda}_{CTC} &= \sum_K \hbar\omega_m \hat{S}^{+}_K \hat{S}^{-}_K + \sum_{k_z>0,k_x} \hbar\omega_{\mathbf{k}} (\hat{a}^{\dagger}_{\mathbf{k}\lambda}\hat{a}_{\mathbf{k}\lambda} + \frac{1}{2}) \\
&- \sum_{k_z>0,k_x} i\sqrt{N}\sqrt{\frac{\hbar c k}{2\varepsilon_0 V}} \Big[\widetilde{\epsilon}_{\mathbf{k}\lambda}(z, x=0)(1+\lambda\overleftrightarrow{\xi}) \cdot \boldsymbol{\mu}_{01} \hat{S}^{+}_{k_x} \hat{a}_{\mathbf{k}\lambda} - h.a.\Big].
\end{aligned} \tag{S41}$$

Comparison with Eq. (S31) clarifies that the chiral effect, i.e., $(1 + \lambda \overset{\leftrightarrow}{\xi})$, remains unchanged. An important difference is that for non-zero in-plane momentum, the coupling is not only to the mode $K_x = 0$ but also to higher momenta, similar to the standard Tavis-Cummings model as shown for example in Ref. [9]

S3 Chiral Hopfield model under the assumption of cancelling instantaneous intermolecular contributions

We provide here a derivation of the chiral Hopfield model under the assumption that the instantaneous intermolecular interactions cancel. Using the explicit form of the chiral fields, our starting point reads then

$$
\begin{aligned}
\hat{H} &= \sum_n \Big[\hat{H}_{M,n} + \frac{1}{2\varepsilon_0 V}(\widetilde{\varepsilon}_k^\lambda(z)\cdot\hat{\boldsymbol{\mu}}_n)^2\Big] \\
&+ \frac{1}{2}\hat{p}_k^2 + \frac{1}{2}\Big[\omega_k^2 + 2\sum_n \hat{\chi}^m_{n,ij}\frac{k^2}{\varepsilon_0 V}\widetilde{\varepsilon}^\lambda_{k,i}(z)\widetilde{\varepsilon}^\lambda_{k,j}(z)\Big]\hat{q}_k^2 \\
&+ \sum_n \sqrt{\frac{1}{\varepsilon_0 V}}\Big[\widetilde{\varepsilon}_k^\lambda(z)\cdot\hat{\boldsymbol{\mu}}_n + \sum_{a,b\in\{x,y,z\}}\hat{Q}_{ab,n}\nabla_{a,n}\widetilde{\varepsilon}^\lambda_{k,b}(z)\Big]\hat{p}_k \\
&- \sum_n \sqrt{\frac{k^2}{\varepsilon_0 V}}\lambda\widetilde{\varepsilon}_k^\lambda(z)\cdot\hat{\mathbf{m}}_n\hat{q}_k
\end{aligned}
\tag{S42}
$$

where the only difference is that the self-polarization is local only.

We can follow the same steps as before and absorb the self-polarization into adjusted local matter frequencies $\widetilde{\omega}_n^2 = \omega_n^2 + \frac{2m\omega_n}{\hbar\varepsilon_0 V}(\widetilde{\varepsilon}_k^\lambda(z)\cdot\boldsymbol{\mu}_{10}^n)^2$ – notice the missing N. We obtain

$$
\begin{aligned}
\hat{H} &= \sum_n \hbar\widetilde{\omega}_n(\hat{b}_n^\dagger\hat{b}_n + \frac{1}{2}) + \hbar\bar{\omega}_k(\hat{a}^\dagger\hat{a} + \frac{1}{2}) \\
&- i\hbar\sum_n \widetilde{g}_n\Big[(\hat{b}_n^\dagger + \hat{b}_n)(\hat{a} - \hat{a}^\dagger) + \widetilde{\xi}_n\lambda(\hat{b}_n^\dagger - \hat{b}_n)(\hat{a} + \hat{a}^\dagger)\Big]
\end{aligned}
\tag{S43}
$$

where as before $\widetilde{g}_n = \sqrt{\frac{\hbar\bar{\omega}_k\omega_n}{2\varepsilon_0 V\widetilde{\omega}_n}}(\boldsymbol{\mu}_{10}^n + \mathbf{Q}_n)\cdot\widetilde{\varepsilon}_k^\lambda(z)$ and $\widetilde{\xi}_n = \frac{\widetilde{\omega}_n\omega_k}{\omega_n\bar{\omega}_k}\frac{\boldsymbol{\mu}_{10}^n\cdot\widetilde{\varepsilon}_k^\lambda(z)}{(\boldsymbol{\mu}_{10}^n + \mathbf{Q}_n)\cdot\widetilde{\varepsilon}_k^\lambda(z)}\xi$ are the renormalized effective coupling strength and chirality factor. Introducing the Fourier-representation leads to

$$
\begin{aligned}
\hat{H} &= \hbar\widetilde{\omega}_m(\hat{B}_{k=0}^\dagger\hat{B}_{k=0} + \frac{1}{2}) + \hbar\widetilde{\omega}_m\sum_{k\neq 0}\hat{B}_k^\dagger\hat{B}_k + (N-1)\frac{\hbar\widetilde{\omega}_m}{2} + 2\hbar\bar{\omega}_k(\hat{a}^\dagger\hat{a} + \frac{1}{2}) \\
&- i\hbar\sqrt{N}\widetilde{g}\Big[(\hat{B}_{k=0}^\dagger + \hat{B}_{k=0})(\hat{a} - \hat{a}^\dagger) + \widetilde{\xi}\lambda(\hat{B}_{k=0}^\dagger - \hat{B}_{k=0})(\hat{a} + \hat{a}^\dagger)\Big]\ .
\end{aligned}
\tag{S44}
$$

Importantly, also the dark states are now dressed by the self-polarization and $\widetilde{\omega}_m$ does not

depend on the number of molecules N. The analytic solution has the same form but deviates in $\tilde{\omega}_m$ which results in instabilities for large N.

Fig. S4 contrasts the two version (left without intersystem self-polarization) of the chiral Hopfield model and the associated instability.

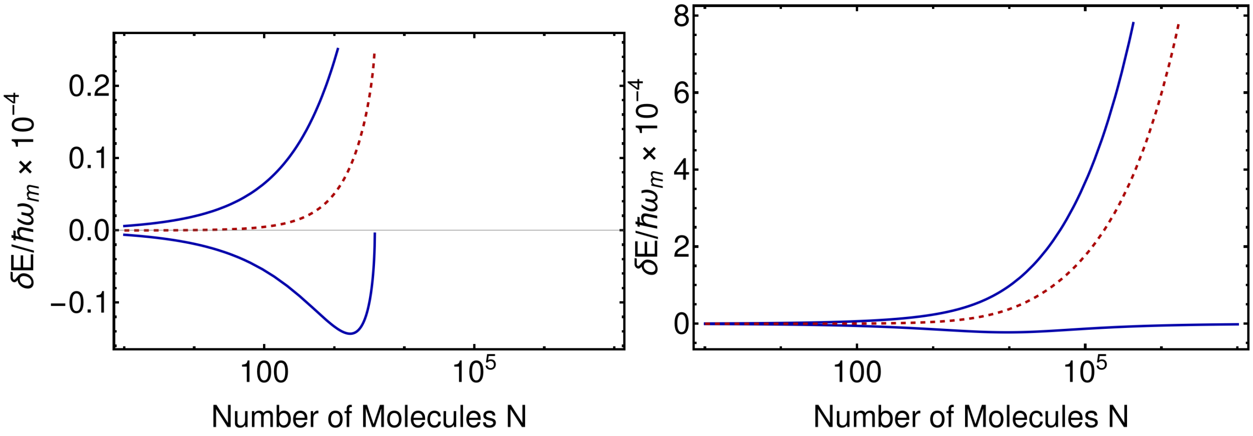

Figure S4: N-scaling differences between left and right-handed chiral dye molecules as introduced in the paper. Left, without intersystem self-polarization, right, using full self-polarization and disregarding intermolecular Coulomb interactions (same as in main text). All parameters consistent with Fig. 3.

S4 Consistency of the Chiral Hopfield model in the ultra-strong coupling domain with *ab intio* calculations

The Hopfield model has been shown to provide excellent results for vibrational strong coupling[10] and intersubband transitions.[7] It is not obvious that the same qualitative accuracy can be expected for the electronic subspace in atomic/molecular structures. However, recent work by Riso et al.[11] utilized an adjusted version of QED Coupled-cluster to estimate the discriminating strength of chiral fields on single and few molecules. They predict a $\sqrt{N}$ behavior of the discriminating strength in the correlated ground-state (Fig. 5 of Ref.[11]) that is consistent with our observations shown in Fig. S5 if the coupling is deep within the ultra-strong coupling domain.

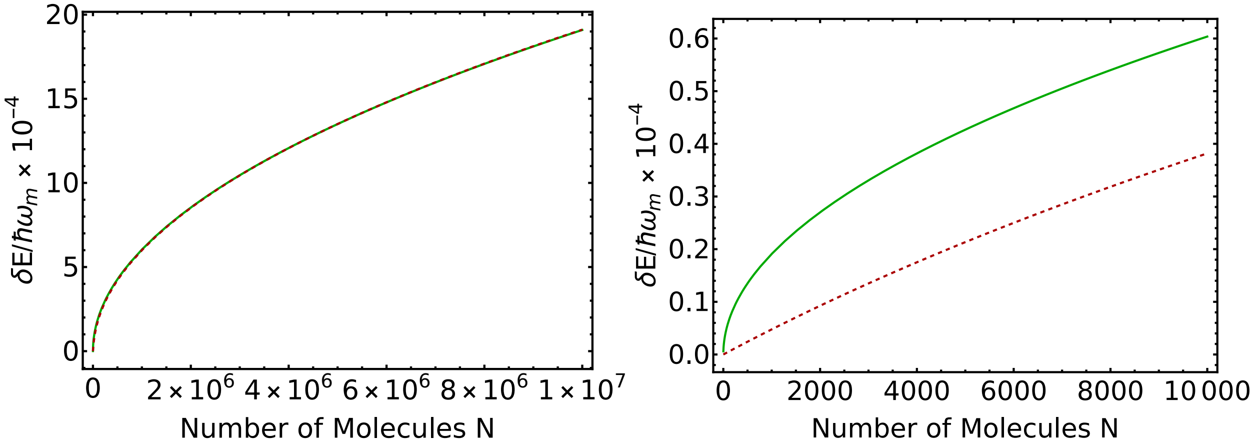

Figure S5: N-scaling differences in the correlated ground-state between left and right-handed chiral dye molecules introduced in the paper (red-dashed). The green line following $6.5 \cdot 10^{-8}\sqrt{N}$ serves as guide to the eye. It is apparent that the large-N limit, which is equivalent with increasing the fundamental coupling strength, is dominated by a $\sqrt{N}$ behavior that is consistent with the available literature. All parameters consistent with Fig. 3.

The overall trend of the polaritonic eigenvalues predicted by the Hopfield model is consistent with exact results for hydrogen as illustrated in Fig. S6.

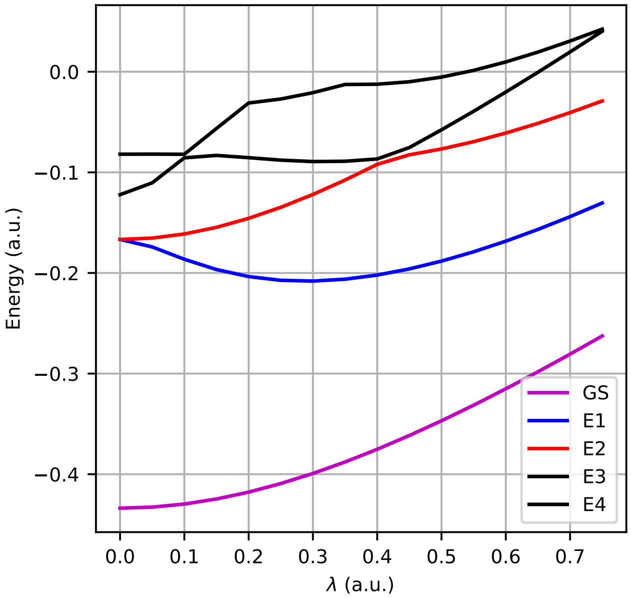

Figure S6: Eigenvalues of two-dimensional soft-Coulomb hydrogen coupled in electric dipole approximation to a single cavity mode in resonance with the first matter-excitation. The exact solution uses a grid representation with 151 x 151 grid-points for hydrogen and 30 Fock-states for the cavity mode. We show the first 5 correlated eigenstates. In total, this amounts to a Hilbert space with 684030 states. Our simplified Hopfield model provides a similar trend (as long as the magnetic components do not dominate the coupling) and is therefore qualitatively consistent with exact solutions. The exact solution will naturally produce (avoided) crossings between higher excited states that are not included in our model but that do not influence the drawn conclusions.

References

(1) Schäfer, C.; Ruggenthaler, M.; Rokaj, V.; Rubio, A. Relevance of the quadratic diamagnetic and self-polarization terms in cavity quantum electrodynamics. *ACS Photonics* **2020**, *7*, 975–990.

(2) Rokaj, V.; Welakuh, D. M.; Ruggenthaler, M.; Rubio, A. Light–matter interaction in the long-wavelength limit: no ground-state without dipole self-energy. *J. Phys. B* **2018**, *51*, 034005.

(3) Condon, E. U. Theories of Optical Rotatory Power. *Reviews of Modern Physics* **1937**, *9*, 432–457.

(4) Corbaton, I. F. Helicity and duality symmetry in light matter interactions: Theory and applications. Ph.D. thesis, Macquarie University, Faculty of Science and Engineering, 2014.

(5) Caloz, C.; Alu, A.; Tretyakov, S.; Sounas, D.; Achouri, K.; Deck-Léger, Z.-L. Electromagnetic nonreciprocity. *Physical Review Applied* **2018**, *10*, 047001.

(6) Hopfield, J. J. Theory of the Contribution of Excitons to the Complex Dielectric Constant of Crystals. *Phys. Rev.* **1958**, *112*, 1555–1567.

(7) Todorov, Y.; Sirtori, C. Intersubband polaritons in the electrical dipole gauge. *Phys. Rev. B* **2012**, *85*, 045304.

(8) Voronin, K.; Taradin, A. S.; Gorkunov, M. V.; Baranov, D. G. Single-handedness chiral optical cavities. *ACS Photonics* **2022**, *9*, 2652–2659.

(9) Tichauer, R. H.; Feist, J.; Groenhof, G. Multi-scale dynamics simulations of molecular polaritons: The effect of multiple cavity modes on polariton relaxation. *The Journal of Chemical Physics* **2021**, *154*, 104112.

(10) George, J.; Chervy, T.; Shalabney, A.; Devaux, E.; Hiura, H.; Genet, C.; Ebbesen, T. W. Multiple Rabi Splittings under Ultrastrong Vibrational Coupling. *Phys. Rev. Lett.* **2016**, *117*, 153601.

(11) Riso, R. R.; Grazioli, L.; Ronca, E.; Giovannini, T.; Koch, H. Strong coupling in chiral cavities: nonperturbative framework for enantiomer discrimination. *arXiv preprint arXiv:2209.01987* **2022**,
