# Supplementary material for: Chiral Polaritonics: Analytical Solutions, Intuition, and Use
Source: J Phys Chem Lett. 2023 Apr 13;14(15):3777–84. doi: 10.1021/acs.jpclett.3c00286 (PMC10123817; doi:10.1021/acs.jpclett.3c00286)
Supplement: Supplementary file 2 — jz3c00286_si_002.pdf [file jz3c00286_si_002.pdf]

jz-2023-00286d.R1

Name: Peer Review Information for "Chiral Polaritonics: Analytic Solutions, Intuition and Its Use"

First Round of Reviewer Comments

Reviewer: 1

Comments to the Author

This is a very interesting letter discussing an as-of-yet underappreciated but timely topic, namely that of chiral polaritonics. In a thorough yet concise manner, the authors present here an analytical quantum-electrodynamical theory in the form of a chiral Tavis–Cummings Hamiltonian, allowing to describe a multitude of chiral quantum emitters interacting with a chiral cavity. In their general discussion they include up to the necessary quadrupole terms, yet for brevity's sake they focus their application on collinear magnetic and electronic transition dipoles. While I have not checked all of their algebra, their results look sensible. As far as I can tell, this is a novel contribution with appropriate interest for publication in the Journal of Physical Chemistry Letters.

I do have a few comments for further improvement of this submission:

1. The authors would do well to make a mentioning of achiral molecules that nevertheless exhibit a chiral optical response, e.g., the non-Sohncke groups, metamaterials, chiroptical interfaces, etc. The authors hint at related ideas in their general formulation of chiroptical activitiy, but then unnecessarily restrict all of their discussion to enantiomers.

2. The authors mention that a high particle number N is necessary for there to be a readily measurable effect for typical molecular dissymmetries. This argument is similar to that proposed for polariton chemistry, where large N values are necessary to benefit from strong coupling effects. With regard to the latter, an ongoing debate is whether a thermodynamic reservoir of "dark" states would mitigate any polaritonic phenomena (in spite of a large Rabi splitting). For a discussion, see for example Dunkelberger et al., Annu. Rev. Phys. Chem. 2022, 73, 429–51. Are the findings by the authors susceptible to a dark state reservoir in much the same way? It would be great if the authors can discuss this.

3. In their discussion the authors justify the investigation by appealing to how chiral polaritons may help us in understanding the origins of homochirality. This may be true, but it ignores the many applied reasons why some might care about this investigation, such as control of chiral reactions as well as spin-related effects. I recommend the authors to discuss this more explicitly.

4. The chiral cavity modes necessary to realize the effects discussed in this paper rely on Fabry–Pérot cavities involving handedness-preserving mirrors, which may be challenging to realize at sufficient finesse/quality factors. I do realize this manuscript is theoretical first and foremost, but it would be good to refer to the experimental state-of-the-art in order to put the findings in a realistic context.

5. On a somewhat related note at point 4, a different branch of chiral polaritonics involves conventional (non-handedness-preserving) Fabry–Pérot cavties combined with quantum emitters with an inverted chiroptical response (relying on 2D chirality rather than 3D chirality). A few relevant papers are:

Salij et al., Phys. Rev. B 2021, 103, 035431

Gautier et al., ACS Photonics 2022, 9, 778–783

Sun et al., Chem. Sci., 2022, 13, 1037-1048

A mentioning of this form of chiral polaritonics would help the reader getting a better grasp of the scope of this topic.

Reviewer: 2

Comments to the Author

In their manuscript, the authors combine molecular polaritonics with the recently proposed chiral cavities. They study the collective interaction of a set of identical chiral molecules with a single standing wave of given circular polarization. Deriving this interaction from the fundamental molecular-QED couplings upon including magnetic terms, they arrive at a Tavis-Cummings-type Hamiltonian which includes an enantiomer-sensitive term. They solve this to find the polaritonic eigen energies of the system which depend on the handedness of the molecules.

The manuscript is extremely well written with a sound and accessible introduction and motivation. The central idea is original and novel, the calculations are well-presented and sound. The result and discussion show that cavity QED has the potential to significantly enhance discriminatory chiral interactions. The manuscript contains a number of intriguing ideas regarding the implications and possible implementation, such as discussions on homochirality or the possibility of designing sub-wavelengh modes with enhanced magnetic components.

I can therefore fully recommend publication and invite the authors to consider the following optional improvements:

- In the introduction, schemes for optical enantiomer separation by Barnett, Genet, and Hornberger are well worth mentioning.

- When establishing the molecular-QED Hamiltonian, the spinless limit is an unnecessary restriction. Spin can be included via a Zeeman-type coupling which will lead to an additional spin component to the magnetic moment that complements the given orbital-angular momentum contribution. The magnetic self-interaction is commonly known as diamagnetic interaction and it may lead to genuine diamagnetic interactions between molecules (which are typically small).

- The chiral cavity could be discussed in slightly more detail. In particular, it would be interesting to learn how the boundary conditions imposed by the mirrors lead to the disappearance of one of the two possible stand-wave polarizations. How is this reduction of the number of modes consistent with the completeness of the Helmholtz operator?

Author's Response to Peer Review Comments:

Dear Prof. Editor,

Thank you for coordinating the review process of our manuscript "Chiral Polaritonics: Analytic Solutions, Intuition and its Us".

We are thankful for the positive evaluation and constructive criticism of our manuscript by all referees. It is reassuring that both authors strongly support our perspective that chiral polaritonics is an important and thus far overlooked topic. We are especially pleased to see that both referee's requested only minor revisions, which we fully accounted for in the revised version, without the need for further review. Please, find below a detailed point-by-point response to all the referees' questions and concerns.

In addition, we adjusted the following formatting aspects as requested:

- added a brief description of the supplementary material
- fixed the style of all references (especially the SI) to use JPCL formatting
- labelled the SI consecutively starting with S1

Christian Schäfer
on behalf of all co-authors
March 8, 2023

Reviewer 1

This is a very interesting letter discussing an as-of-yet underappreciated but timely topic, namely that of chiral polaritonics. In a thorough yet concise manner, the authors present here an analytical quantum-electrodynamical theory in the form of a chiral Tavis–Cummings Hamiltonian, allowing to describe a multitude of chiral quantum emitters interacting with a chiral cavity. In their general discussion they include up to the necessary quadrupole terms, yet for brevity's sake they focus their application on collinear magnetic and electronic transition dipoles. While I have not checked all of their algebra, their results look sensible. As far as I can tell, this is a novel contribution with appropriate interest for publication in the Journal of Physical Chemistry Letters.

We thank the referee for a very positive and constructive evaluation of our manuscript and fully agree that chiral polaritonics is a promising new direction that requires more attention.

Comment 1. *The authors would do well to make a mentioning of achiral molecules that nevertheless exhibit a chiral optical response, e.g., the non-Sohncke groups, metamaterials, chiroptical interfaces, etc. The authors hint at related ideas in their general formulation of chiroptical activitiy, but then unnecessarily restrict all of their discussion to enantiomers.*

Our reply: We thank the referee for raising our awareness for this unfortunate miscommunication from our side. Indeed, our work requires merely a chiral resonant optical scatterer, which may be present in the form of a molecular enantiomer or a geometrically chiral meta-atom made of achiral medium. We have included a paragraph clarifying that the result of our work also apply to resonant chiral plasmonic meta-atoms and metasurfaces.

Comment 2. *The authors mention that a high particle number N is necessary for there to be a readily measurable effect for typical molecular dissymmetries. This argument is similar to that proposed for polariton chemistry, where large N values are necessary to benefit from strong coupling effects. With regard to the latter, an ongoing debate is whether a thermodynamic reservoir of "dark" states would mitigate any polaritonic phenomena (in spite of a large Rabi splitting). For a discussion, see for example Dunkelberger et al., Annu. Rev. Phys. Chem. 2022, 73, 429–51. Are the findings by the authors susceptible to a dark state reservoir in much the same way? It would be great if the authors can discuss this.*

Our reply: Our work emphasizes two conclusions to be drawn from the chiral models: 1) the Rabi-splitting depends on the handedness of the chiral optical response of the emitter in relation to the cavity, 2) the correlated ground-state energy is affected in essentially the same way. The excitation energies of the dark states remain unchanged and both effects are limited by the rather small chirality of existing molecules. Systems with stronger chirality would allow for a much stronger control, which

might be simply due to another matter system or the assistance of sub-wavelength resonators. As pointed out by the referee, if only 2 bright states compete against N-1 dark states, one would naively expect practically no change of any internal process for $N \longrightarrow \infty$. Aspect (1) of our work is affected by the dark states in precisely the same way as polaritonic chemistry. To all our surprise, this does not seem to be the case in experimental reality. In contrast, sizeable changes in the chemical reactivity have been measured when molecules are subjected to a resonant cavity. The community is unfortunately missing a conclusive theoretical explanation for this conundrum and our publication does not claim to add to this discussion. In our work, polaritonic chemistry serves as an unknown mechanism that merely requires collective strong coupling as input to deliver a handedness depending change in the reactivity and thermodynamic characteristics. Our work demonstrates that chiral cavities are able to create selective strong coupling, i.e., the mechanism behind polaritonic chemistry will result in different reactivity for different enantiomers. Chiral polaritonics should be seen as a filter that is able to selectively switch the mechanism behind polaritonic chemistry. The previous version attempted to clarify this perspective from the start, i.e., the first 2 sentences of the abstract emphasize that cavity QED is a tool to control chemical reactivity. However, we fully support the referee's comment and decided to emphasize this aspect in the main text and conclusion.

Comment 3. *In their discussion the authors justify the investigation by appealing to how chiral polaritons may help us in understanding the origins of homochirality. This may be true, but it ignores the many applied reasons why some might care about this investigation, such as control of chiral reactions as well as spin-related effects. I recommend the authors to discuss this more explicitly.*

Our reply: Our introduction sets a strong focus on the practical importance of chiral discrimination for chemistry, biology, and medicine. We agree that this aspect is important. However, we also raise awareness that the rather small intrinsic chirality will currently result in very small effects in the pratical discrimination, as discussed in detail on page 6 and 7. We discuss options to enhance this effect. In conclusion, we expect the handedness depending coupling to be experimentally measured and our conclusions to be validated, but do not expect a decisive change in reactivity without the use of more sophisticated designs. On the other hand, homochirality is build around the 'smallness' of the coupling, i.e., a small effect of chiral polaritonics would be actually desirable in this case and a direct use can be drawn. We emphasize this now stronger in the discussion/conclusion.

Comment 4. *The chiral cavity modes necessary to realize the effects discussed in this paper rely on Fabry–Pérot cavities involving handedness-preserving mirrors, which may be challenging to realize at sufficient finesse/quality factors. I do realize this manuscript is theoretical first and foremost, but it would be good to refer to the experimental state-of-the-art in order to put the findings in a realistic context.*

Our reply: Although single-handedness chiral cavities have not been yet realized, there is a substantial

progress in the design and experimental characterization of handedness-preserving mirrors, which are an essential part of such a cavity. A brief discussion and reference to Semnani et al. (Light: Science and Applications 2020, 9, 23.) has been added to the revised manuscript.

Comment 5. *On a somewhat related note at point 4, a different branch of chiral polaritonics involves conventional (non-handedness-preserving) Fabry–Pérot cavties combined with quantum emitters with an inverted chiroptical response (relying on 2D chirality rather than 3D chirality). A few relevant papers are:*
Salij et al., Phys. Rev. B 2021, 103, 035431
Gautier et al., ACS Photonics 2022, 9, 778–783
Sun et al., Chem. Sci., 2022, 13, 1037-1048
A mentioning of this form of chiral polaritonics would help the reader getting a better grasp of the scope of this topic.

Our reply: We thank the referee for pointing our attention to these papers. We have added Salij et al. to the introduction as circularly-polarized quantum emitters (such as valley excitons) do not fit the scope of chirality defined in our publication. Furthermore, we added a brief comment regarding 2D chirality (Gautier et al.) and Faraday systems (Sun et al.) as examples for alternative directions.

Reviewer 2

In their manuscript, the authors combine molecular polaritonics with the recently proposed chiral cavities. They study the collective interaction of a set of identical chiral molecules with a single standing wave of given circular polarization. Deriving this interaction from the fundamental molecular-QED couplings upon including magnetic terms, they arrive at a Tavis-Cummings-type Hamiltonian which includes an enantiomer-sensitive term. They solve this to find the polaritonic eigen energies of the system which depend on the handedness of the molecules.
The manuscript is extremely well written with a sound and accessible introduction and motivation. The central idea is original and novel, the calculations are well-presented and sound. The result and discussion show that cavity QED has the potential to significantly enhance discriminatory chiral interactions. The manuscript contains a number of intriguing ideas regarding the implications and possible implementation, such as discussions on homochirality or the possibility of designing sub-wavelengh modes with enhanced magnetic components.

We thank the referee for this extremely positive and constructive evaluation of our manuscript.

Comment 1. *In the introduction, schemes for optical enantiomer separation by Barnett, Genet, and Hornberger are well worth mentioning.*

Our reply: We thank the referee for drawing our attention to these works. We have mentioned two new references discussing optical-force assisted separation of enantiomers (Cameron et al., New Journal of Physics 2014, 16, 013020 and Genet et al., ACS photonics 2022, 9, 319).

Comment 2. *When establishing the molecular-QED Hamiltonian, the spinless limit is an unnecessary restriction. Spin can be included via a Zeeman-type coupling which will lead to an additional spin component to the magnetic moment that complements the given orbital-angular momentum contribution. The magnetic self-interaction is commonly known as diamagnetic interaction and it may lead to genuine diamagnetic interactions between molecules (which are typically small).*

Our reply: Our initial goal was to provide a fully consistent theory on the lowest level of nonrelativistic QED but we agree with the referee that removing the spin is not necessary. The latter may be either derived from Dirac's equation or added heuristically. The revised version accounts now for the spin in compact form.

Comment 3. *The chiral cavity could be discussed in slightly more detail. In particular, it would be interesting to learn how the boundary conditions imposed by the mirrors lead to the disappearance of one of the two possible stand-wave polarizations. How is this reduction of the number of modes consistent with the completeness of the Helmholtz operator?*

Our reply: Assuming ideal mirrors for simplicity, the matching handedness (lets say RH) is perfectly confined with the common characteristics of idealized FP-cavities. The other handedness (LH then) is unaffected by the mirrors, i.e., the cavity is transparent to LH modes. The interaction of molecules in the cavity is therefore strong with the RH modes but weak with the LH free-spaces modes. Strong coupling, i.e., Tavis-Cummings and Hopfiled, descriptions appear therefore only with the confined RH modes while the LH modes could be accounted for by a bath description. We emphasize this aspect better in the revised manuscript when introducing the mode quantization (see p. 4).

We thank both referee's for the extraordinarily constructive and positive report and are confident that the revised version accounts for all comments.
